# Probing the Unruh effect with an accelerated extended system

**DOI:** 10.1038/s41467-019-10962-y

**Published:** 2019-07-10

**Authors:** Cesar A. Uliana Lima, Frederico Brito, José A. Hoyos, Daniel A. Turolla Vanzella

**Affiliations:** Instituto de Física de São Carlos, Universidade de São Paulo, Caixa Postal 369, São Carlos, 13560-970 São Paulo Brazil

**Keywords:** Quantum information, Theoretical physics, Statistical physics

## Abstract

It has been proved in the context of quantum fields in Minkowski spacetime that the vacuum state is a thermal state according to uniformly accelerated observers—a seminal result known as the Unruh effect. Recent claims, however, have challenged the validity of this result for extended systems, thus casting doubts on its physical reality. Here, we study the dynamics of an extended system, uniformly accelerated in the vacuum. We show that its reduced density matrix evolves to a Gibbs thermal state with local temperature given by the Unruh temperature $T_{\mathrm{U}} = \hbar a/(2\pi ck_{\mathrm{B}})$, where *a* is the system’s spatial-dependent proper acceleration—*c* is the speed of light and *k*_B_ and $\hbar$ are the Boltzmann’s and the reduced Planck’s constants, respectively. This proves that the vacuum state does induce thermalization of an accelerated extended system—which is all one can expect of a legitimate thermal reservoir.

## Abstract (web summary)

The Unruh effect is a seminal result which illustrates the relative character of the particle concept in QFT, but its validity for extended systems is debated. Here, the authors show how a uniformly accelerated extended system of two coupled spins will evolve to a Gibbs thermal state at the Unruh temperature.

## Introduction

Soon after Hawking published his seminal result on particle creation due to black hole formation, leading to the phenomenon of black hole evaporation^1^, Unruh clarified the relative character of the particle concept in the context of quantum field theory in flat spacetime. More specifically, he showed that the vacuum state—which represents the absence of particles according to inertial observers—corresponds to a thermal bath with temperature *T*_U_ = ℏ*a*/(2*πck*_B_) for uniformly accelerated observers^2^, where *a* is the observers’ proper acceleration (ℏ is the reduced Planck’s constant, *c* is the speed of light, and *k*_B_ is the Boltzmann’s constant). This result has become known as the Unruh effect (see ref. ^3^ for a comprehensive review). Although some deep connections may be established between Hawking’s result and the Unruh effect—and, in fact, the former served as motivation for Unruh’s analysis—the latter is not as well known as the former. This is somewhat unfortunate because some conceptual issues raised by black hole evaporation can be better understood through the lens of Unruh’s result—e.g., that the possibility of Hawking radiation being in a mixed state does not violate any quantum principle (therefore, no information-loss “paradox” is present^4^). But even among those who are acquainted with the Unruh effect, not rarely it is misinterpreted as saying that the thermal bath experienced by accelerated observers in the vacuum state of a quantum field would be indistinguishable from a thermal state of the same field at temperature *T* = *T*_U_ according to inertial observers—which is a false statement. This overly stringent view has compelled many to challenge or restrict the validity of the Unruh effect based on non-local observables which behave differently in these two situations. Simple examples of such observables are two-point correlation functions^5^. Also, the fact that uniformly accelerated observers, static with respect to each other, can have different proper accelerations *a* (depending on their separation), makes the Unruh temperature *T*_U_ spatially inhomogeneous across the uniformly accelerated frame. This peculiar behavior has been held against the interpretation of *T*_U_ as a physical temperature when spatially-extended systems are considered^6^. Although having no analogue for an inertial (equilibrium) thermal state, this inhomogeneity of *T*_*U*_ is mandatory in the accelerated frame. This is a direct, well-known consequence of relativistic redshift effects^7^, as we shall discuss later.

Our main goal here is to settle this debate by providing convincing evidence that the strict thermal nature of the Minkowski vacuum in the uniformly accelerated frame is physically meaningful also for spatially-extended systems—which sense non-local observables and inhomogeneous *T*_U_—upholding *T*_U_ as a legitimate temperature. In order to achieve this, we analyze a system composed of two uniformly accelerated spins, separated by an arbitrary finite distance *d* (fixed in their accelerated rest frame), directly coupled to each other—which confers unity to the system—and locally coupled to quantum fields in the vacuum state. We focus attention on the reduced density matrix of the accelerated-spins’ system and show that it evolves to an equilibrium state which, according to an arbitrary observer with proper acceleration *a* static with the spins, is exactly the one which would be expected if the system were in contact with a thermal bath with temperature *T* = *T*_U_; in other words, we show that the spin system thermalizes at a nonzero, well-defined temperature due to the vacuum fluctuations it experiences in its accelerated rest frame. This corroborates the view that although one might construct observables which distinguish the Unruh thermal bath from an ordinary (i.e., inertial) one at the same temperature—and, in fact, we show that the thermalization time scales can distinguish these two situations, which, we stress, is not in conflict with the Unruh effect—the former does act as a legitimate thermal reservoir also for extended systems.

## Results

### The setup

Unless stated otherwise, we adopt natural units, in which ℏ = *c* = *k*_B_ = 1. Let us consider two spin-1/2 point particles, A and B, whose spins **s**_A_ and **s**_B_ are directly coupled to each other via, say, the (free) Hamiltonian $\hat H_0 = - J\hat s_{\mathrm{A}}^{\mathrm{z}}\hat s_{\mathrm{B}}^{\mathrm{z}}$, *J* ≠ 0, which has two-fold degenerate eigenvalues ±*J*/4. Since the spins are taken to be spatially separated, this is a simple, yet legitimate model of an extended system. Now, let us couple (locally and weakly) the spins to a quantum field, such that neither an eventual constant of motion would prevent the spin system from thermalizing, nor the dynamics could be mapped onto a two free-particle problem. As a matter of fact, the simplest spin-field interaction which would lead to some interesting evolution is given by linearly coupling one of the other spin components, say $\hat s_{{\mathrm{A}}({\mathrm{B}})}^{\mathrm{x}}$, to a massless, scalar quantum field ${\hat{\mathrm{\Phi }}}$. However, this would lead to a conservation law for the observable $\hat s_{\mathrm{A}}^{\mathrm{x}}\hat s_{\mathrm{B}}^{\mathrm{x}}$, which, in turn, would split the state space of the spin system as if it were two noninteracting (non-localized) spins. In order to avoid such a symmetry, we shall couple the other spins’ component, $\hat s_{{\mathrm{A}}({\mathrm{B}})}^{\mathrm{y}}$, to another massless, scalar field, so that the (time-dependent) Hamiltonian is given by:

1 $$\hat H(\tau ) = - J\hat s_{\mathrm{A}}^{\mathrm{z}}\hat s_{\mathrm{B}}^{\mathrm{z}} + q\mathop {\sum }\limits_{j \in \{ {\mathrm{x}},{\mathrm{y}}\} } \,\left[ {\frac{{{\hat{\mathrm{\Phi }}}_{\mathrm{A}}^j(\tau )\hat s_{\mathrm{A}}^j}}{{u_{\mathrm{A}}^0(\tau )}} + \frac{{{\hat{\mathrm{\Phi }}}_{\mathrm{B}}^j(\tau )\hat s_{\mathrm{B}}^j}}{{u_{\mathrm{B}}^0(\tau )}}} \right],$$

where $q \in {\Bbb R}$ is a dimensionless (scalar) coupling constant, ${\hat{\mathrm{\Phi }}}_{{\mathrm{A}}({\mathrm{B}})}^j(\tau ): = {\hat{\mathrm{\Phi }}}^j(\tau ,{\mathbf{x}}_{{\mathrm{A}}({\mathrm{B}})}(\tau ))$ are massless, scalar quantum fields—which are independent for different *j* ∈ {x, y}—evaluated at the spins’ location **x**_A(B)_(*τ*), and $u_{{\mathrm{A}}({\mathrm{B}})}^0: = d\tau /d\tau _{{\mathrm{A}}({\mathrm{B}})}$ is the time component of the four-velocity of spin A(B), with *τ*_A(B)_ being its proper time. Note that there is no need to include the free Hamiltonian of the fields ${\hat{\mathrm{\Phi }}}^j$ in Eq. (1) since we already take into account the explicit time-dependence of ${\hat{\mathrm{\Phi }}}^j$ enforced by the free-field Klein–Gordon equation, $\square{\hat{\mathrm{\Phi }}}^j = 0$. The coordinate system {(*τ*, **x**)} would be arbitrary at this point. However, since we are going to consider that the system evolves according to the von Neumann equation (Eq. (2) below), it is necessary that *τ* represents the parameter of a time-translation symmetry of the spacetime—∂/∂*τ* is a time-like Killing field—and that $\hat H$ given in Eq. (1) is the Hamiltonian of the system in the (stationary) reference frame associated with this symmetry. For instance, if the spins were static in an inertial frame, then *τ* could be conveniently set to be the usual inertial time—for which $u_{{\mathrm{A}}({\mathrm{B}})}^0 = 1$. For accelerated spins which are static in a uniformly accelerated frame, as we are interested here, *τ* can be interpreted as the proper time of a fiducial uniformly accelerated observer with respect to (w.r.t.) whom the spins are static. The presence of $u_{{\mathrm{A}}({\mathrm{B}})}^0$ in the interaction terms accounts for the fact that $\hat H$ evolves the system in the time parameter *τ*, while $q\,{\hat{\mathrm{\Phi }}}_{{\mathrm{A}}({\mathrm{B}})}\hat s_{{\mathrm{A}}({\mathrm{B}})}^j$, being a local interaction, is related to the evolution in the time parameter *τ*_A(B)_. In particular, care must be taken when interpreting the meaning of the parameter *J*: it is twice the energy gap Δ*E* of the spin system as measured by the fiducial observer. According to an observer at spin-A(B) location, this gap is inevitably corrected by the “redshift” factor $u_{{\mathrm{A}}({\mathrm{B}})}^0$: ${\mathrm{\Delta }}E_{{\mathrm{A}}({\mathrm{B}})} = u_{{\mathrm{A}}({\mathrm{B}})}^0{\mathrm{\Delta }}E$. Without any loss of generality, the fiducial observer can be placed at, say, spin-A’s position, so that *τ* = *τ*_A_—which leads to $u_{\mathrm{A}}^0 = 1$. As we shall see later, the value of $u_{\mathrm{B}}^0$ depends on whether the acceleration of the system is perpendicular or parallel to its spatial extension. (The fact that ${\mathrm{\Delta }}E_{\mathrm{A}}/{\mathrm{\Delta }}E_{\mathrm{B}} = u_{\mathrm{A}}^0/u_{\mathrm{B}}^0$—which is independent of *J* and of the choice of fiducial observer—plays an essencial role in interpreting our results in the parallel case, in which our extended system feels an inhomogeneous Unruh temperature.) The model defined by Eq. (1) can be considered as an extension of the well-known spin-boson model, which is taken as a paradigm for the study of the dissipative dynamics of two-level systems^8^.

Let $\hat \rho$ be the positive semidefinite, Hermitian, trace-class operator (with trace 1) describing the state of the whole universe (spins + fields). Its evolution is governed by

2 $${\mathrm{i}}\partial _\tau \hat \rho = [\hat H(\tau ),\hat \rho ],$$

whose solution can be written as

3 $$\hat \rho (\tau ) = \hat U(\tau ,\tau _0)\hat \rho _0[\hat U(\tau ,\tau _0)]^{ - 1},$$

with $\hat \rho _0: = \hat \rho (\tau _0)$ and $\hat U(\tau ,\tau _0)$ satisfying

4 $${\mathrm{i}}\partial _\tau \hat U(\tau ,\tau _0) = \hat H(\tau )\hat U(\tau ,\tau _0),\quad \hat U(\tau _0,\tau _0) = \hat 1.$$

We are only interested in the reduced density matrix of the spin system, obtained after tracing out the fields’ degrees of freedom (system’s reduced matrix):

5 $$\hat \rho _{\mathrm{s}}(\tau ): = {\mathrm{tr}}_{\mathrm{\Phi }}\left[ {\hat \rho (\tau )} \right].$$

Motived by the results obtained from the spin-boson model^8^, we shall treat the coupling with the quantum fields as a (time-dependent) perturbation, $\hat V(\tau ): = q\mathop {\sum }\limits_{j \in \{ {\mathrm{x}},{\mathrm{y}}\} } \,[{\hat{\mathrm{\Phi }}}_{\mathrm{A}}^j(\tau )\hat s_{\mathrm{A}}^j/u_{\mathrm{A}}^0(\tau ) + {\hat{\mathrm{\Phi }}}_{\mathrm{B}}^j(\tau )\hat s_{\mathrm{B}}^j/u_{\mathrm{B}}^0(\tau )]$, on the free Hamiltonian $\hat H_0 = - J\hat s_{\mathrm{A}}^{\mathrm{z}}\hat s_{\mathrm{B}}^{\mathrm{z}}$. Indeed, under this regime, namely, the weak coupling regime, the spin-boson model provides means for observing spin thermalization process, with predicted decoherence/relaxation time scales matching those observed in physical systems satisfying the conditions imposed. For that, we write $\hat U(\tau ,\tau _0) = {\mathrm{e}}^{ - {\mathrm{i}}\hat H_0(\tau - \tau _0)}\hat U_{\mathrm{I}}(\tau ,\tau _0)$, where $\hat U_{\mathrm{I}}$ represents the time evolution operator in the interaction picture, satisfying

6 $${\mathrm{i}}\partial _\tau \hat U_{\mathrm{I}}(\tau ,\tau _0) = \hat H_{\mathrm{I}}(\tau )\hat U_{\mathrm{I}}(\tau ,\tau _0),\quad \hat U_{\mathrm{I}}(\tau _0,\tau _0) = \hat 1,$$

with

7 $${\hat H_{\mathrm{I}}(\tau ): = {\mathrm{e}}^{{\mathrm{i}}\hat H_0{\mathrm{\Delta }}\tau }\hat V(\tau ){\mathrm{e}}^{ - {\mathrm{i}}\hat H_0{\mathrm{\Delta }}\tau } = q\mathop {\sum }\limits_{j \in \{ {\mathrm{x}},{\mathrm{y}}\} } \,\left[ {\frac{{{\hat{\mathrm{\Phi }}}_{\mathrm{A}}^j(\tau )\hat s_{\mathrm{A}}^j(\tau )}}{{u_{\mathrm{A}}^0(\tau )}} + \frac{{{\hat{\mathrm{\Phi }}}_{\mathrm{B}}^j(\tau )\hat s_{\mathrm{B}}^j(\tau )}}{{u_{\mathrm{B}}^0(\tau )}}} \right],}$$

where Δ*τ* := *τ* − *τ*_0_ and

8 $$\hat s_M^j(\tau ) = \hat s_M^j\,{\mathrm{cos}}\left( {\frac{{J{\mathrm{\Delta }}\tau }}{2}} \right) + 2{\mathrm{i}}\left[ {\hat s_M^j,\hat s_M^{\mathrm{z}}} \right]\hat s_{\bar M}^{\mathrm{z}}\,{\mathrm{sin}}\left( {\frac{{J{\mathrm{\Delta }}\tau }}{2}} \right),$$

with *M* ∈ {A, B} and $${\bar{\mathrm{A}}}: = {\mathrm{B}}$$, $${\bar{\mathrm{B}}}: = {\mathrm{A}}$$.

Solving Eq. (6) iteratively (as a Dyson series), working consistently up to second order in *q*, and restricting attention to the case where the initial state is simply separable, $\hat \rho _0 = \hat \rho _{{\mathrm{s}}0} \otimes \hat \rho _{{\mathrm{\Phi }}0}$, where $\hat \rho _{{\mathrm{s}}0}$ and $\hat \rho _{{\mathrm{\Phi }}0}$ describe the initial state of the spin system and of the fields, respectively, we obtain, from Eq. (5):

9 $${\begin{array}{*{20}{l}} {\hat \rho _{\mathrm{s}}(\tau )} \hfill & = \hfill & {{\mathrm{e}}^{ - {\mathrm{i}}\hat H_0{\mathrm{\Delta }}\tau }\Big\{ {\hat \rho _{{\mathrm{s}}0} - \frac{{q^2}}{2}\mathop {\sum }\limits_{M,N \in \{ {\mathrm{A}},{\mathrm{B}}\} } \,\mathop {\smallint }\limits_{\tau _0}^\tau \,\frac{{d\tau^\prime }}{{u_M^0(\tau^\prime )}}\,\mathop {\smallint }\limits_{\tau _0}^\tau \,\frac{{d\tau^{\prime\prime} }}{{u_N^0(\tau^{\prime\prime} )}}\mathop {\sum }\limits_{j \in \{ {\mathrm{x}},{\mathrm{y}}\} } \,{\mathrm{i}}G_{\mathrm{F}}^j(x_M^\prime ,x_N^{\prime\prime} )} } \hfill \\ {} \hfill & {} \hfill & { {{\mathrm{T}}\left\{ {\left[ {\hat s_M^j(\tau^\prime ),\hat s_N^j(\tau^{\prime\prime} )\hat \rho _{{\mathrm{s}}0}} \right]} \right\} + {\mathrm{H}}.{\mathrm{c}}.} \Big\}\,{\mathrm{e}}^{{\mathrm{i}}\hat H_0{\mathrm{\Delta }}\tau } + {\cal{O}}(q^3),} \hfill \end{array}}$$

where H.c. stands for the Hermitian conjugate of the term which precedes it and ${\mathrm{i}}G_{\mathrm{F}}^j(x^\prime ,x^{\prime\prime}): = {\mathrm{tr}}_{\mathrm{\Phi }}\left\{ {\hat \rho _{{\mathrm{\Phi }}0}{\mathrm{T}}\left[ {{\hat{\mathrm{\Phi }}}^j(x^{\prime}){\hat{\mathrm{\Phi }}}^j(x^{\prime\prime})} \right]} \right\}$ are the time-ordered Feynman correlators in state $\hat \rho _{{\mathrm{\Phi }}0}$. (The usual time-ordering operator T appearing explicitly in the second line of Eq. (9) must be applied before the commutator is expanded.) Since we are interested only in the effects of quantum fluctuations of ${\hat{\mathrm{\Phi }}}^j$ on the spin system, we have already assumed $\langle {\mathrm{\Phi }}^j(x)\rangle : = {\mathrm{tr}}_{\mathrm{\Phi }} \left\{ {\hat \rho _{{\mathrm{\Phi }}0}{\hat{\mathrm{\Phi }}}^j(x)} \right\} = 0$, which, together with the independence of ${\hat{\mathrm{\Phi }}}^j$ for different *j*, implies ${\mathrm{tr}}_{\mathrm{\Phi }}\left\{ {\hat \rho _{{\mathrm{\Phi }}0}{\hat{\mathrm{\Phi }}}^{\mathrm{x}}(x^{\prime}){\hat{\mathrm{\Phi }}}^{\mathrm{y}}(x^{\prime\prime})} \right\} = 0$. Also, we restrict attention to the case where ${\mathrm{i}}G_{\mathrm{F}}^{\mathrm{x}}(x^{\prime},x^{\prime\prime}) \equiv {\mathrm{i}}G_{\mathrm{F}}^{\mathrm{y}}(x^{\prime},x^{\prime\prime}) = :{\mathrm{i}}G_{\mathrm{F}}(x^{\prime},x^{\prime\prime})$, which applies to the situation in which we are most interested.

### Static spins’ arrangements in static field states

Restricting attention to static spins’ arrangements *x*_A_, *x*_B_ and static field states $\hat \rho _{{\mathrm{\Phi }}0}$ (w.r.t. the time parameter *τ*), it follows that *G*_F_(*x*_*M*_′, *x*_*N*_″) can depend on *τ*′ and *τ*″ only through the combination *ξ* := *τ*′ − *τ*″, *G*_F_(*x*_*M*_′, *x*_*N*_″) =: *G*_*MN*_(*ξ*)—in addition to $u_M^0(\tau ) \equiv u_M^0$ being constant. This suggests that it may be more convenient, in the second-order term of Eq. (9), to perform a change of integration variables to *η* := (*τ*′ + *τ*″)/2 and *ξ*. Notice that, by construction, *G*_*MN*_(*ξ*) = *G*_*NM*_(−*ξ*), which, in particular, implies that *G*_AA_(*ξ*) and *G*_BB_(*ξ*) are even distributions w.r.t. *ξ*. But staticity also implies that *G*_AB_(*ξ*) and *G*_BA_(*ξ*) are even distributions w.r.t. *ξ*; hence, *G*_AB_(*ξ*) ≡ *G*_BA_(*ξ*). Using Eq. (8) into Eq. (9), the integral in *η* can be explicitly evaluated, leading to:

10 $${\begin{array}{*{20}{l}} {{\mathrm{e}}^{{\mathrm{i}}\hat H_0{\mathrm{\Delta }}\tau }\hat \rho _{\mathrm{s}}(\tau ){\mathrm{e}}^{ - {\mathrm{i}}\hat H_0{\mathrm{\Delta }}\tau }} \hfill & = \hfill & {\hat \rho _{{\mathrm{s}}0} - \frac{{q^2}}{2}\mathop {\sum }\limits_{M,N \in \{ {\mathrm{A}},{\mathrm{B}}\} } \,\mathop {\smallint }\nolimits_{ - {\mathrm{\Delta }}\tau }^{{\mathrm{\Delta }}\tau } \,d\xi \,\frac{{{\mathrm{i}}G_{MN}(\xi )}}{{u_M^0u_N^0}}\left\{ {\left( {{\mathrm{\Delta }}\tau - |\xi |} \right)\left[ {\hat C_{(MN)}^{( + )}\,{\mathrm{cos}}\left( {\frac{{J\xi }}{2}} \right)} \right.} \right.} \hfill \\ {} \hfill & {} \hfill & {\left. { + \,\hat D_{(MN)}^{( - )}\,{\mathrm{sin}}\left( {\frac{{J|\xi |}}{2}} \right)} \right] + \frac{2}{J}{\mathrm{sin}}\left( {\frac{{J({\mathrm{\Delta }}\tau - |\xi |)}}{2}} \right)\left[ {\hat C_{(MN)}^{( - )}{\mathrm{cos}}\left( {\frac{{J{\mathrm{\Delta }}\tau }}{2}} \right)} \right.} \hfill \\ {} \hfill & {} \hfill & {\left. {\left. { + \,\hat D_{(MN)}^{( + )}\,{\mathrm{sin}}\left( {\frac{{J{\mathrm{\Delta }}\tau }}{2}} \right)} \right]} \right\} + {\mathrm{H}}.{\mathrm{c}}. + {\cal{O}}(q^3),} \hfill \end{array}}$$

where

11 $$\hat C_{MN}^{( \pm )}: = \mathop {\sum }\limits_{j \in \{ {\mathrm{x}},{\mathrm{y}}\} } \,\left\{ {\frac{1}{2}\left[ {\hat s_M^j,\hat s_N^j\hat \rho _{s0}} \right] \pm 2\left[ {\hat s_M^{\bar j}\hat s_{\bar M}^{\mathrm{z}},\hat s_N^{\bar j}\hat s_{\bar N}^{\mathrm{z}}\hat \rho _{{\mathrm{s}}0}} \right]} \right\},$$

12 $$\hat D_{MN}^{( \pm )}: = \mathop {\sum }\limits_{j \in \{ {\mathrm{x}},{\mathrm{y}}\} } \,\varepsilon _{j\bar j}\left\{ {\left[ {\hat s_M^{\bar j}\hat s_{\bar M}^{\mathrm{z}},\hat s_N^j\hat \rho _{{\mathrm{s}}0}} \right] \pm \left[ {\hat s_M^j,\hat s_N^{\bar j}\hat s_{\bar N}^{\mathrm{z}}\hat \rho _{{\mathrm{s}}0}} \right]} \right\},$$

with ${\bar{\mathrm{x}}}: = {\mathrm{y}}$, ${\bar{\mathrm{y}}}: = {\mathrm{x}}$, $\varepsilon _{{\mathrm{xy}}} = - \varepsilon _{{\mathrm{yx}}} = {\mathrm{ }}1$, and indices *M*, *N* inside parentheses in Eq. (10) denotes symmetrization: *X*_(*MN*)_ := (*X*_*MN*_ + *X*_*NM*_)/2.

As it stands, Eq. (10), being a truncated perturbative expansion, is not appropriate to investigate long-term features of the spin system, as relaxation to an eventual equilibrium state when Δ*τ* → ∞. In this limit, the second-order term in *q* is, in general, unbounded and, therefore, cannot be consistently considered as providing a “small” deviation from the free evolution. We can, nonetheless, try to break long-term evolution into a sequence of *N* (≫1) time lapses Δ*τ* such that in each time lapse, for sufficiently small coupling *q*, the spins’ evolution is well described by Eq. (10). This strategy is trivially valid for closed systems. Here, however, since tracing out the fields’ degrees of freedom at the end of each time step does not necessarily lead to the same result as taking the trace only after *N* steps, this procedure is not guaranteed, in general, to lead to the correct evolution of the reduced density matrix. Notwithstanding this, in the Methods’ subsection “Validity of the Markovian regime,” we show that there is a finite time-lapse scale Δ*τ* (≫*J*^−1^, ∥**x**_A_ − **x**_B_∥) for which this strategy holds true—resembling a Markovian regime—leading to the long-term evolution

13 $$\hat \rho _{\mathrm{s}}(\tau _N) = {\mathrm{e}}^{ - {\mathrm{i}}\hat H_0\tau _N}\left( {{\mathrm{e}}^{ - q^2{\mathrm{R}}_0\tau _N}\hat \rho _{{\mathrm{s}}0}} \right){\mathrm{e}}^{{\mathrm{i}}\hat H_0\tau _N},$$

where *τ*_*N*_ = *N*Δ*τ* and ${\mathrm{R}}_0:{\cal{T}}\left( {{\cal{H}}_{\mathrm{s}}} \right) \to {\cal{T}}\left( {{\cal{H}}_{\mathrm{s}}} \right)$—an operator acting on the space of trace-class operators describing the spin system—is determined by the equation [recall Eqs. (11) and (12)]

14 $${{\mathrm{R}}_0\left( {\hat \rho _{\mathrm{s}}} \right) = \frac{1}{2}\mathop {\sum }\limits_{M,N \in \{ {\mathrm{A}},{\mathrm{B}}\} } \,\left. {\left\{ {{\mathrm{i}}\pi \tilde G_{MN}(J/2)\hat C_{(MN)}^{( + )} - P_{J/2}\left[ {{\mathrm{i}}\tilde G_{MN}} \right]\hat D_{(MN)}^{( - )}} \right\}} \right|_{\hat \rho _{{\mathrm{s}}0} \mapsto \hat \rho _{\mathrm{s}}} + {\mathrm{H.c.}},}$$

with

15 $$\tilde G_{MN}(\omega ): = \frac{1}{{2\pi }}\mathop {\smallint }\limits_{ - \infty }^\infty \,d\xi \,\frac{{G_{MN}(\xi )}}{{u_M^0u_N^0}}\,{\mathrm{e}}^{{\mathrm{i}}\omega \xi }$$

and

16 $${\cal{P}}_a[f]: = \lim_{\epsilon \to 0}\mathop {\smallint }\limits_{{\Bbb R}\backslash [ - \epsilon ,\epsilon ]} \,\frac{{f(x + a)}}{x}\,dx.$$

In the Methods’ subsection “Decay modes and related decay rates of the spin system,” we present all eigenvalues *λ*_*k*_ and (right) eigenvectors (or “eigenmatrices”) $\hat \rho _k$ of R_0_ (with *k* = 1, …, 16) in terms of ${\mathrm{i}}\tilde G_{MN}(J/2)$, ${\cal{P}}_{J/2}\left[ {{\mathrm{i}}\tilde G_{MN}} \right]$, and the elements of the Bell basis $\{ |{\mathrm{\Psi }}_{{\mathrm{AB}}}^{( \pm )}\rangle ,|{\mathrm{\Phi }}_{{\mathrm{AB}}}^{( \pm )}\rangle \}$ defined in Eqs. (31) and (32)—see Eqs. (35)–(62); note that each individual mode $\hat \rho _k$ does not necessarily have to represent a physical state. This encodes complete information about the evolution of the spin system. It is a simple task to verify that these eigenmatrices $\hat \rho _k$ are also eigenmatrices of the free evolution: ${\mathrm{E}}_0(\hat \rho _k): = {\mathrm{e}}^{ - {\mathrm{i}}\hat H_0{\mathrm{\Delta }}\tau }\hat \rho _k{\mathrm{e}}^{{\mathrm{i}}\hat H_0{\mathrm{\Delta }}\tau } = {\mathrm{e}}^{ - {\mathrm{i}}E_k{\mathrm{\Delta }}\tau }\hat \rho _k$, where, depending on *k*, *E*_*k*_ = 0, ±*J*/2. Therefore, noticing that *λ*_1_ = *E*_1_ = 0 and provided Re(*λ*_*k*≠1_) > 0—as will be verified later in our cases of interest—we finally obtain the evolution of the spin system in the Markovian regime:

17 $$\hat \rho _{\mathrm{s}}(\tau _N) = \mathop {\sum }\limits_{k = 1}^{16} \,c_k{\mathrm{e}}^{ - (q^2\lambda _k + {\mathrm{i}}E_k)\tau _N}\hat \rho _k\mathop { \to }\limits^{N \to \infty } \hat \rho _1 = :\hat \rho _{{\mathrm{eq}}},$$

where the coefficients *c*_*k*_ are uniquely determined by the initial condition $\mathop {\sum }\limits_{k = 1}^{16} \,c_k\hat \rho _k = \hat \rho _{{\mathrm{s}}0}$—in particular, $c_1 = {\mathrm{tr}}_{\mathrm{s}}\left\{ {\hat \rho _{{\mathrm{s}}0}} \right\} = 1$.

### Uniformly accelerated spins in the vacuum

Finally, in this section we apply the expressions obtained above to our case of interest: uniformly accelerated spins in the vacuum. The vacuum state |0〉 of a free, massless scalar field is characterized as being the (unique) Poincaré-invariant state of the theory. The vacuum expectation value of the field vanishes, $\langle 0|{\hat{\mathrm{\Phi }}}(x)|0\rangle = 0$, whereas its two-point (Wightman) function is given by

18 $$W(x,x^\prime ): = \langle 0|{\hat{\mathrm{\Phi }}}(x){\hat{\mathrm{\Phi }}}(x^\prime )|0\rangle = \frac{1}{{4\pi ^2\sigma _\epsilon (x,x^\prime )}},$$

where $\sigma _\epsilon \left( {x,x^\prime } \right)$ is the ($\epsilon$-regularized) square of the geodesic “distance” between events *x* and *x*′—which is obtained from the square of the geodesic distance, *σ*(*x*, *x*′), by introducing an infinitesimal negative imaginary part (−i$\epsilon$) into the time coordinate of the first event *x*. As expected from its definition, notice that *W*(*x*, *x*′) is a bi-scalar. Therefore, its value is insensitive to the choice of coordinate system we use to represent the events *x* and *x*′. In terms of inertial Cartesian coordinates {(*t*, *X*, *Y*, *Z*)}, *σ*(*x*, *x*′) = −(*t* − *t*′)^2^ + (*X* − *X*′)^2^ + (*Y* − *Y*′)^2^ + (*Z* − *Z*′)^2^, whereas in terms of coordinates {(*τ*, *X*, *Y*, *ζ*)} well adapted to a uniformly accelerated frame—defined through *t* = (*ζ* + *a*^−1^)sinh(*aτ*), *Z* = (*ζ* + *a*^−1^)cosh(*aτ*), with *ζ* > −*a*^−1^, $\tau \in {\Bbb R}$—we have

19 $$\sigma (x,x^\prime ) =	 - \frac{4}{{a^2}}(a\zeta + 1)(a\zeta^\prime + 1)\left[ {{\mathrm{sinh}}\left( {\frac{{a(\tau - \tau^\prime )}}{2}} \right)} \right]^2 \\ 	+ (X - X^\prime )^2 + (Y - Y^\prime )^2 + (\zeta - \zeta^\prime )^2,$$

where *a* > 0 is a constant. The interpretation of *τ* and *ζ* follows from the form of the Minkowski line element in these coordinates,

20 $$ds^2 = - (1 + a\zeta )^2d\tau ^2 + dX^2 + dY^2 + d\zeta ^2:$$

the coordinate *τ* is the proper time of (fiducial) observers static at *ζ* = 0—whose constant proper acceleration is given by the parameter *a*—whereas the coordinate *ζ* measures spatial distances along the acceleration direction, according to observers static in this coordinate system. Notice [for the sake of the discussion below Eq. (1)] that *τ* does represent a time-translation symmetry of the spacetime. This can be inferred from the line element given in Eq. (20), since the coefficients of the differentials (i.e., the metric components) are independent of *τ*—∂/∂*τ* is a time-like Killing field known as the boost Killing field.

**Fig. 1:**
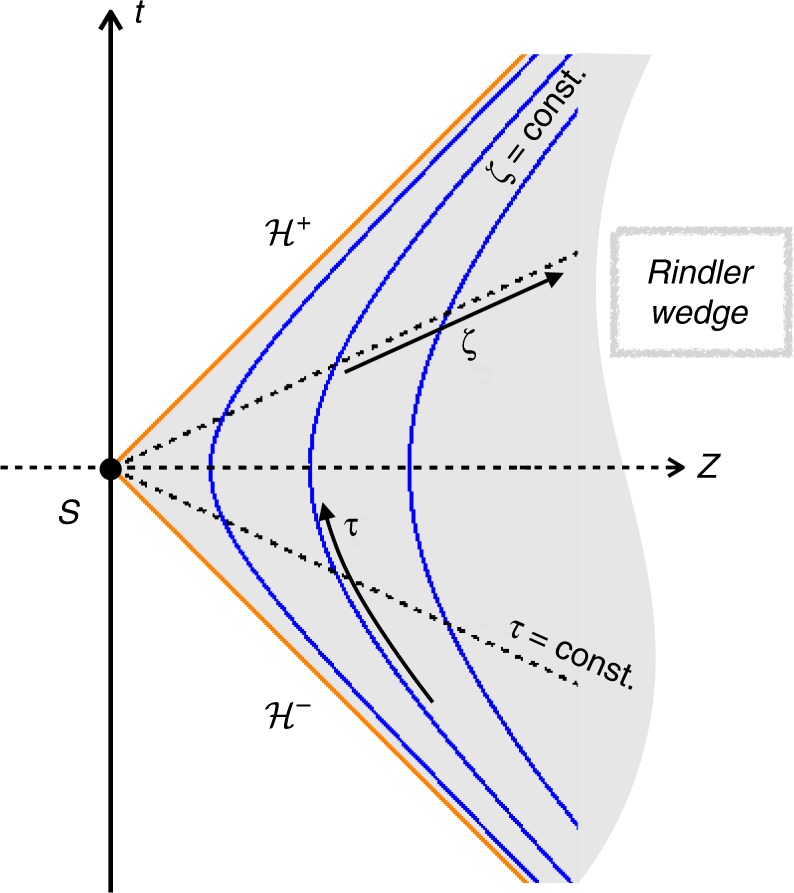
Uniformly accelerated frame and the Rindler wedge. Depiction of the Minkowski spacetime region *Z* > |*t*| covered by the coordinates *τ* and *ζ* (suppressing coordinates *X* and *Y*). This is commonly referred to as the Rindler wedge. Solid (blue) curves represent the (*ζ* = constant) worldlines of uniformly-accelerated observers “at rest” w.r.t. each other, while dashed (black) lines represent *τ* = constant hypersurfaces—which encodes simultaneity according to these observers. All constant-*ζ* curves asymptote ${{\cal{H}}^+}$ and ${\cal{H}}^-$, reflecting that this uniformly-accelerated frame does not cover the whole spacetime. Moreover, there is a spacetime region which is causally inaccessible to observers in this frame, for whom ${\cal{H}}^+$ represents a (future) event horizon

For the sake of completeness, let us recall some basic facts about accelerated frames which are relevant for our purposes. Figure 1 presents a diagram depicting the Minkowski spacetime region *Z* > |*t*| (called Rindler wedge) which is covered by the accelerated Cartesian coordinates {(*τ*, *X*, *Y*, *ζ*)} defined above Eq. (19) (suppressing the *X* and *Y* directions). In such a diagram, worldlines of inertial observers would be represented by straight lines making an angle smaller than 45° with the vertical *t* axis, while light rays are represented by 45° lines. Constant-*ζ* (hyperbolic) curves represent worldlines of a family of uniformly-accelerated observers “at rest” w.r.t. each other—which constitutes a uniformly-accelerated frame. All these hyperbolas (for given *X* and *Y*) asymptote the same light rays (on ${{\cal{H}}^+}$ and ${{\cal{H}}^-}$) which intersect at *S* (a flat 2-surface when *X* and *Y* are restored). This shows that, contrary to inertial frames, no single uniformly-accelerated frame can cover the whole (Minkowski) spacetime. Moreover, to any uniformly-accelerated observer, there is a causally inaccessible spacetime region, with ${{\cal{H}}^+}$ playing the role of an event horizon. This particular feature is essencial in understanding how a pure quantum state (the Minkowski vacuum) is perceived as a mixed (thermal) state by uniformly-accelerated observers. In terminology introduced in ref. ^9^, the Unruh thermal bath is an example of an improper mixed state, arising from tracing out some degrees of freedom of a pure state—the ones inaccessible to the accelerated observers. In contrast, inertial thermal states are truly statistical mixtures of pure states (energy-momentum eigenstates according to inertial observers), representing the whole system—which qualifies them as proper mixed states. Although this kind of distinction may be relevant on a conceptual level—see, e.g., ref. ^10^ for a discussion in the context of information loss in black hole evaporation—it plays no role for our purposes since observables restricted to the Rindler wedge cannot uncover the improper nature of the Unruh thermal bath.

The fact that constant-*ζ* curves have common asymptotes—instead of simply being translated hyperbolas in the *Z* direction—also shows that uniformly-accelerated observers static at different values of *ζ* have different proper accelerations. In fact, it can be shown that *ζ* = constant worldlines have proper acceleration given by *a*_*ζ*_ = *a*/(1 + *aζ*)—and it follows directly from Eq. (20) that observers following these worldlines have proper time given by *τ*_*ζ*_ = (1 + *aζ*)*τ*. Thus, according to the Unruh effect, each such observer “feels” a different Unruh temperature *T*_U_ = *a*_*ζ*_/(2*π*). More concretely, a uniformly-accelerated extended system (with constant proper spatial dimensions) would “feel” an inhomogeneous Unruh temperature along the direction of its acceleration. This inhomogeneity—which our simple model for an extended system can probe—is at the heart of the arguments against *T*_U_ being a legitimate physical temperature^6^. Note, however, that $T_{\mathrm{U}}\sqrt { - g_{00}} =$ constant—*g*_00_ being the time-time metric component read from Eq. (20)—evidencing the role played by the redshift effect in the spatial variation of *T*_U_. This constraint, $T\sqrt { - g_{00}} =$ constant, is known as Tolman’s relation^7^ and is valid for thermal states in arbitrary (flat and curved) stationary spacetimes—for it follows simply from the condition that the net heat flow in an equilibrium state must vanish everywhere.

It is worth pointing out that all this discussion about uniformly-accelerated frames, event horizons, and the Unruh effect is only relevant for interpreting our final results, not for carrying out any of the calculations. As far as the calculations are concerned, all we need is the vacuum two-point function *W*(*x*, *x*′), given by Eq. (18), and information about the spins’ worldlines. The use of *τ* and *ζ* instead of *t* and *Z* to express the bi-scalar *σ*(*x*, *x*′)—in Eq. (19)—can be seen as a mere mathematical convenience, since uniformly-accelerated worldlines separated by a constant proper distance take the simple form *ζ* = constant. In addition, nowhere in the calculations we express the (pure) Minkowski vacuum state as a thermal bath of (Rindler) particles according to uniformly accelerated observers—i.e., we do not assume the Unruh effect to be true. From our perspective, we are inertial theorists using standard quantum theory to predict the behavior of an extended “thermometer” accelerating in the vacuum.

### Spins with equal proper accelerations

Let us first consider the case where the spins are uniformly accelerated perpendicularly to their spatial separation, with the same proper acceleration *a*. This can be described, in the coordinates {(*τ*, *X*, *Y*, *ζ*)}, by spin trajectories given by *X*_A_(*τ*) ≡ *Y*_A_(*τ*) ≡ *ζ*_A_(*τ*) ≡ *Y*_B_(*τ*) ≡ *ζ*_B_(*τ*) ≡ 0, *X*_B_(*τ*) ≡ *d*, where *d* is the spatial separation between the spins (in their rest frame) and, conveniently, *τ* ≡ *τ*_A_ ≡ *τ*_B_ (i.e., $u_{\mathrm{A}}^0 = u_{\mathrm{B}}^0 = 1$). This, combined with the Wightman function given in Eq. (18), is all we need to calculate the eigenvalues *λ*_*k*_ and eigenmatrices $\hat \rho _k$ appearing in the long-term evolution of the spin system [Eq. (17)], as presented in more detail in the Methods’ subsection “Transformed Feynman correlators and their principal values”.

**Fig. 2:**
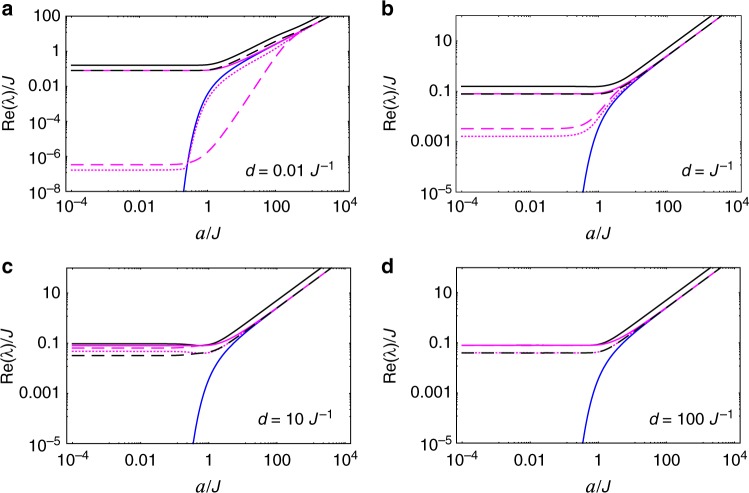
Acceleration dependence of relaxation/decoherence rates of the spin system with equal proper accelerations. We plot the decay rates (normalized by *q*^2^), Re(*λ*_*k*_)—*k* = 2 (solid, black line), 3 (dashed, magenta line), 4, 5, 6 (solid, blue line), 7, 8, 9, 10 (dotted, magenta line), 11, 12, 13, 14 (dashed, black line), 15, 16 (solid, magenta line)—of the decaying modes of the spin system, as functions of the spins’ acceleration *a*, for different separations *d*. Unless *d* ≪ *J*^−1^—in which case mode $\hat \rho _3$ dominates the late-time dynamics for $J \lesssim a \lesssim d^{ - 1}$—modes $\hat \rho _4,\hat \rho _5,\hat \rho _6$ (all belonging to the lowest-energy subspace) dictate how the system approaches equilibrium with a relaxation/decoherence rate given by *q*^2^*J*/[8*π*(e^*πJ*/*a*^ − 1)]

**Fig. 3:**
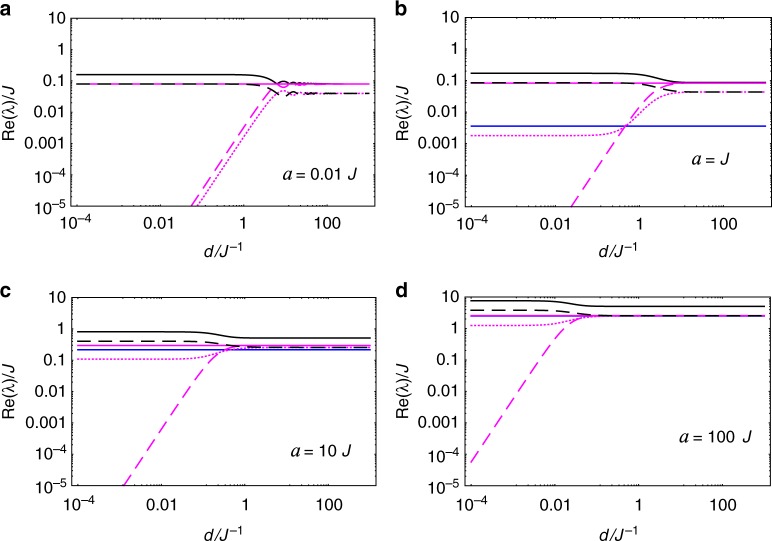
Spin-separation dependence of relaxation/decoherence rates of the spin system with equal proper accelerations. We plot the decay rates (normalized by *q*^2^), Re(*λ*_*k*_) (using same style code as in Fig. 2), of the modes $\hat \rho _k$, as functions of the distance *d* between the spins, for different accelerations *a*. Note that for any *a* > 0, the relaxation rate of mode $\hat \rho _3$ goes to zero as *d* → 0

The nonzero eigenvalues *λ*_*k*_ (*k* = 2, …, 16) are related, through Eq. (17), to the decoherence/relaxation rates of the spin system. In Fig. 2, we plot all these rates (normalized by *q*^2^) as functions of the proper acceleration *a*, for different separations *d*. In case $d \gtrsim J^{ - 1}$, there are basically two regimes of acceleration: *a* ≪ *J*—in which modes $\hat \rho _4,\hat \rho _5,\hat \rho _6$ (all belonging to the lowest-energy subspace) dictate how the system approaches equilibrium with a relaxation/decoherence rate given by *q*^2^*J*e^−*πJ*/*a*^/(8*π*)—and *a* ≫ *J*—in which all relaxation/decoherence rates degenerate in just two values: *q*^2^*a*/(8*π*^2^) and twice this value. Such a result, namely, a relaxation rate that scales as a power law of the temperature—recall that *T*_U_ = *a*/(2*π*)—is known to be a signature of certain inertial baths in the limit of high temperatures. Indeed, for the class of baths known as Ohmic environments, it is precisely established for the spin-boson model that the decay rate will have a power law dependence, which is linear in a second order system-bath coupling perturbation theory^8^. In case *d* ≪ *J*^−1^, there appears a third, moderate regime of acceleration ($J \lesssim a \lesssim d^{ - 1}$) in which mode $\hat \rho _3$ dictates how equilibrium is approached, with a relaxation/decoherence rate given approximately by *q*^2^*Jd*^2^*a*^2^/(12*π*). In Fig. 3, we plot the same decoherence/relaxation rates, now as functions of the spins’ separation *d*, for different values of proper acceleration *a*.

From Eq. (17), we see that the accelerated spin system eventually evolves to an equilibrium state $\hat \rho _{{\mathrm{eq}}} = \hat \rho _1$ given by Eq. (43). Using Eqs. (65)–(69), this equilibrium state, written in terms of the elements of the Bell basis $\{ |{\mathrm{\Psi }}_{{\mathrm{AB}}}^{( \pm )}\rangle ,|{\mathrm{\Phi }}_{{\mathrm{AB}}}^{( \pm )}\rangle \}$, reads

21 $$\hat \rho _{{\mathrm{eq}}} = \frac{1}{{\cal{Z}}}\left[ {\mathrm{e}}^{\pi J/(2a)}\left( {|{\mathrm{\Psi }}_{{\mathrm{AB}}}^{( + )}\rangle \langle {\mathrm{\Psi }}_{{\mathrm{AB}}}^{( + )}| + |{\mathrm{\Psi }}_{{\mathrm{AB}}}^{( - )}\rangle \langle {\mathrm{\Psi }}_{{\mathrm{AB}}}^{( - )}|} \right)\right. \\ \left. + {\mathrm{e}}^{ - \pi J/(2a)}\left( {|{\mathrm{\Phi }}_{{\mathrm{AB}}}^{( + )}\rangle \langle {\mathrm{\Phi }}_{{\mathrm{AB}}}^{( + )}| + |{\mathrm{\Phi }}_{{\mathrm{AB}}}^{( - )}\rangle \langle {\mathrm{\Phi }}_{{\mathrm{AB}}}^{( - )}|} \right) \right]=\frac{{{\mathrm{e}}^{ - \beta \hat H_0}}}{\cal{Z}},$$

where ${\cal{Z}}: = 2\left[ {{\mathrm{e}}^{\pi J/(2a)} + {\mathrm{e}}^{ - \pi J/(2a)}} \right] = {\mathrm{tr}}_{\mathrm{s}}({\mathrm{e}}^{ - \beta \hat H_0})$ and *β* := 2*π*/*a*. In other words, for any initial state $\hat \rho _{{\mathrm{s}}0}$, the final equilibrium state $\hat \rho _{{\mathrm{e}}q}$ of the spin system is the Gibbs thermal state with temperature *β*^−1^ = *a*/(2*π*), the Unruh temperature. (Observe that $|{\mathrm{\Psi }}_{{\mathrm{AB}}}^{( + )}\rangle \langle {\mathrm{\Psi }}_{{\mathrm{AB}}}^{( + )}| + |{\mathrm{\Psi }}_{{\mathrm{AB}}}^{( - )}\rangle \langle {\mathrm{\Psi }}_{{\mathrm{AB}}}^{( - )}|$ and $|{\mathrm{\Phi }}_{{\mathrm{AB}}}^{( + )}\rangle \langle {\mathrm{\Phi }}_{{\mathrm{AB}}}^{( + )}| + |{\mathrm{\Phi }}_{{\mathrm{AB}}}^{( - )}\rangle \langle {\mathrm{\Phi }}_{{\mathrm{AB}}}^{( - )}|$ are the corresponding identity matrices for the energy eigenvalue subspaces −*J*/4 and *J*/4, meaning that, as expected, the thermalization process does not favor any of the possible eigenstates of those subspaces.) The spin system thermalizes due to the vacuum fluctuations it experiences in its accelerated frame—despite these fluctuations being distinct from the ones in inertial thermal states, as properly noted in ref. ^5^—vindicating the Unruh effect also for an extended system.

### Spins with different proper accelerations

Now, we consider the spatial separation *d* of the two spins to be along the direction of their accelerations: *X*_A_(*τ*) ≡ *Y*_A_(*τ*) ≡ *ζ*_A_(*τ*) ≡ *X*_B_(*τ*) ≡ *Y*_B_(*τ*) ≡ 0, *ζ*_B_(*τ*) ≡ *d*. In this case, *τ* ≡ *τ*_A_ ≡ *τ*_B_/(1 + *ad*) (i.e., $u_{\mathrm{A}}^0 = 1$ and $u_{\mathrm{B}}^0 = 1/(1 + ad)$) and *a* continues to be the proper acceleration of spin A, while spin-B proper acceleration is given by *a*_B_ = *a*/(1 + *ad*) [recall discussion on uniformly-accelerated frames after Eq. (20)]. Therefore, according to the Unruh effect, each spin sees a different local temperature at its position.

**Fig. 4:**
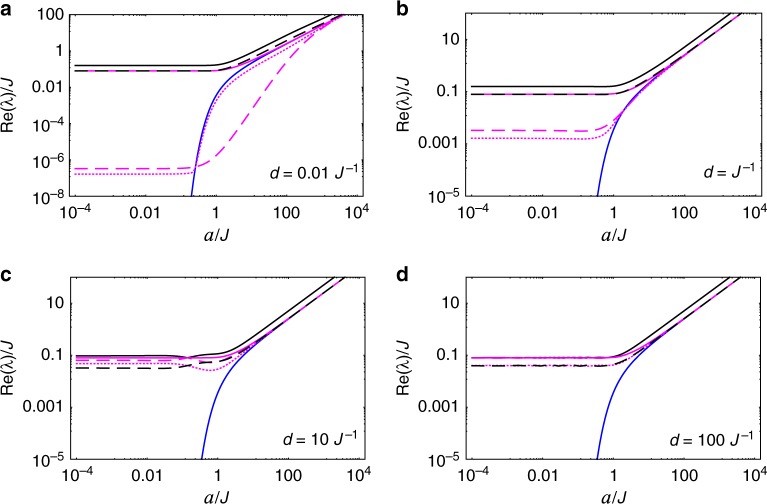
Acceleration dependence of relaxation/decoherence rates of the spin system with different proper accelerations. We plot the decay rates (normalized by *q*^2^ and measured by observers at spin-A location), Re(*λ*_*k*_), of the decaying modes of the spin system, as functions of the spin-A acceleration *a*, for different separations *d*. We use the same style code as in Fig. 2, to which it is almost identical

**Fig. 5:**
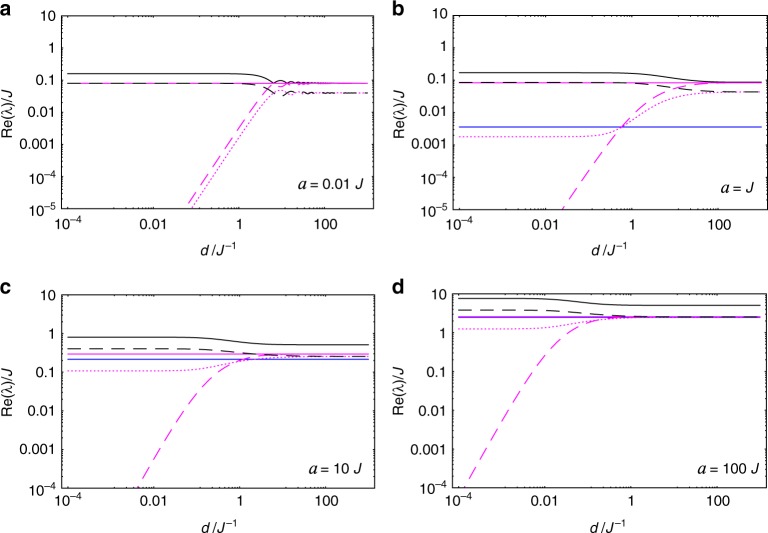
Spin-separation dependence of relaxation/decoherence rates of the spin system with different proper accelerations. We plot the decay rates (normalized by *q*^2^ and measured by observers at spin-A location), Re(*λ*_*k*_) (using same style code as in Fig. 2), of the modes $\hat \rho _k$, as functions of the distance *d* between the spins, for different spin-A accelerations *a*. Again, note the extreme similarity with Fig. 3

**Fig. 6:**
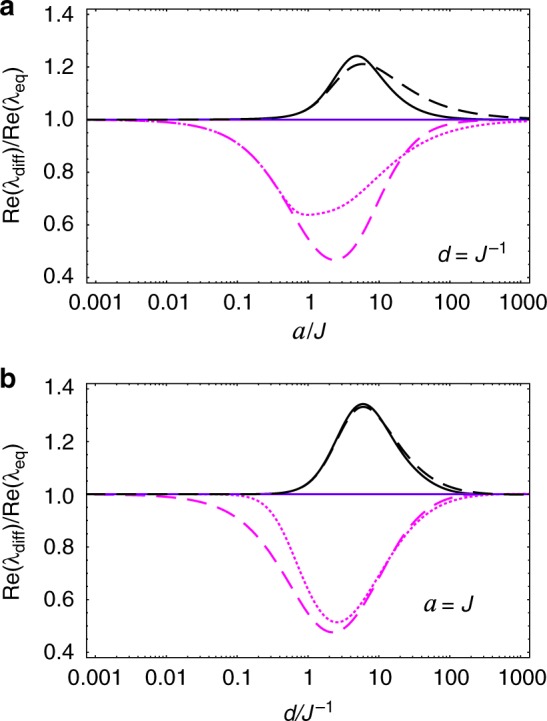
Ratio between the relaxation/decoherence rates for different scenarios. For each decaying mode $\hat \rho _k$ of the spin system, we plot (using the same style code as in Fig. 2) the ratios Re(*λ*_diff_)/Re(*λ*_eq_), where Re(*λ*_diff_) is the decay rate in the case of spins with different proper accelerations and Re(*λ*_eq_) is the decay rate in the case of spins with equal proper accelerations. In (**a**) the ratios are plotted as functions of spin-A’s proper acceleration *a* (for fixed *d* = *J*^−1^), while in (**b**) the ratios are plotted as functions of the spins’ separation *d* (for fixed *a* = *J*)

Following the same steps of the previous, equal-acceleration case, we determine *λ*_*k*_ and $\hat \rho _k$ which govern the long-term evolution of the spin system for different proper accelerations—see Methods’ subsection “Transformed Feynman correlators and their principal values.” It turns out that the overall behavior in this case is very similar to the case of equal accelerations. This can be readily seen from Figs. 4 and 5—where we plot the relaxation/decoherence rates (as measured by observers static at *ζ* = 0 and normalized by *q*^2^) of the spin system as functions of the (spin-A) proper acceleration *a* and as functions of the separation *d*, respectively—which should be compared with Figs. 2 and 3. Note that the corresponding figures are almost identical, making the previous discussion on the behavior of the relaxation/decoherence rates for *a*_A_ = *a*_B_ also valid for this scenario where *a*_A_ ≠ *a*_B_. In order to better visualize the effect of the unequal proper accelerations on the spin system, we plot in Fig. 6 the ratio between the relaxation/decoherence rates for different accelerations, Re(*λ*_diff_), and for equal accelerations, Re(*λ*_eq_), for each decaying mode of the reduced density matrix. We see that intermediate values of *ad* lead to maximum differences between these two scenarios.

Most important for our purposes, though, is the fact that, as in the case with *a*_A_ = *a*_B_, the final equilibrium state $\hat \rho _1$ takes the form of the Gibbs state given by Eq. (21); i.e., the spin system thermalizes at a temperature *β*^−1^ = *a*/(2*π*) according to observers at *ζ* = 0—for whom $\hat H_0$ is the free Hamiltonian of the extended system. As for observers at *ζ* ≠ 0 (e.g., with spin B, *ζ* = *d*)—for whom the proper time is $\tilde \tau = (1 + a\zeta )\tau$—the same Gibbs state describes thermal equilibrium at temperature *T*_U_(*ζ*) = *a*/[2*π*(1 + *aζ*)] = *a*_*ζ*_/(2*π*), since, for them, the Hamiltonian of the spin system [i.e., the time-evolution operator appearing in Eq. (2) with *τ* substituted by $\tilde \tau /(1 + a\zeta )$ is given by $\hat H_0/(1 + a\zeta )$. Although the observed local temperatures are different—ensuring no net heat flow across the system, as pointed out earlier—the same final state given by Eq. (21) is a true thermal equilibrium state for all observers simultaneously,

22 $$\hat \rho _{{\mathrm{eq}}} \propto {\mathrm{exp}}\left( { - \frac{{2\pi }}{a}\hat H_0} \right) = {\mathrm{exp}}\left( { - \frac{{2\pi }}{{a_\zeta }}\frac{{\hat H_0}}{{(1 + a\zeta )}}} \right),$$

once more corroborating the physical reality of the Unruh thermal bath and its inhomogeneous temperature for an extended system.

## Discussion

We made use of an accelerated extended system (two directly coupled spins, each of which linearly and weakly coupled to quantum fields in the vacuum state) in order to show that the standard interpretation of the Unruh effect—that the inertial vacuum state acts as a legitimate thermal reservoir according to uniformly accelerated observers—is strictly correct even when considering extended systems. It is indeed true, as pointed out by ref. ^5^, that correlations seen by uniformly-accelerated observers in the vacuum state differ from the ones seen by inertial observers in an inertial thermal state. For instance, the vacuum two-point function evaluated along two uniformly-accelerated worldlines, in the simpler case where they have the same proper acceleration *a* (perpendicular to their separation *d*), reads [see Eqs. (18) and (64)]

23 $$W_{{\mathrm{ac}}}^ \bot (\tau ,d) = - \frac{1}{{4\pi ^2}}\frac{{a^2}}{{\left\{ {4\left[ {{\mathrm{sinh}}\left( {a\tau /2 - {\mathrm{i}}\epsilon } \right)} \right]^2 - a^2d^2} \right\}}},$$

whereas the two-point function evaluated along two inertial wordlines at rest in an inertial thermal state with temperature *T* is given by^11^

24 $${W_{{\mathrm{in}}}^{(T)}(\tau ,d) = \frac{T}{{8\pi d}}\left[ {{\mathrm{coth}}\left( {\pi T(\tau + d - {\mathrm{i}}\epsilon )} \right) - {\mathrm{coth}}\left( {\pi T(\tau - d - {\mathrm{i}}\epsilon )} \right)} \right],}$$

with *τ* and *d* being, in both expressions, the proper-time difference and proper distance, respectively, measured by observers following the corresponding worldlines. It is not difficult to verify that there is no correspondence between *a* and *T* which makes these two expressions equal (as functions of *τ* and *d*), except in the limit case *d* → 0, for which $W_{{\mathrm{ac}}}^ \bot (\tau ,0) \equiv W_{{\mathrm{in}}}^{(T_{\mathrm{U}})}(\tau ,d \to 0)$, *T*_U_ = *a*/(2*π*). This also occurs in the case of uniformly-accelerated worldlines with different proper accelerations, where the vacuum two-point function evaluated along these worldlines reads [see Eqs. (18) and (71)]

25 $$W_{{\mathrm{ac}}}^\parallel (\tau ,d) = - \frac{1}{{4\pi ^2}}\frac{{a^2}}{{\left\{ {4(1 + ad)\left[ {{\mathrm{sinh}}\left( {a\tau /2 - {\mathrm{i}}\epsilon } \right)} \right]^2 - a^2d^2} \right\}}}.$$

Again, $W_{{\mathrm{ac}}}^\parallel (\tau ,d)$ ≢ $W_{{\mathrm{in}}}^{(T)}(\tau ,d)$ for any fixed relation between *T* and *a*, but $W_{{\mathrm{ac}}}^\parallel (\tau ,0) \equiv W_{{\mathrm{in}}}^{(T_{\mathrm{U}})}(\tau ,d \to 0)$, *T*_U_ = *a*/(2*π*). This means that, although point-like probes cannot distinguish between (i) being with constant proper acceleration *a* in the vacuum and (ii) being at rest in an inertial thermal bath with temperature *T* = *T*_U_, extended probes can. In fact, our Fig. 6 illustrates this well: while spatially-extended probes at rest in an inertial thermal bath cannot exhibit any dependence on its spatial orientation, the decoherence/relaxation time scales of our extended system do depend on the system’s orientation w.r.t. its acceleration—owning to the fact that $W_{{\mathrm{ac}}}^\parallel (\tau ,d)$ ≢ $W_{{\mathrm{ac}}}^ \bot (\tau ,d)$.

This kind of behavior has been occasionally interpreted as a violation of the strict thermal character of the Unruh effect for extended systems and non-local observables. However—and this is the important point—this distinct behavior in situations (i) and (ii) is not in conflict with the rigorous statement of the Unruh effect, which only says that the vacuum state is a thermal state according to uniformly-accelerated observers. Thermal states at the same temperature need not be equal, for they depend on the Hamiltonian describing the system; and the Hamiltonian carries a subtle but important dependence on the family of observers w.r.t. whom the time evolution is being considered. Putting it more clearly: the Hamiltonian of a quantum field according to a family of inertial observers is not the same as the one according to a family of uniformly-accelerated observers; therefore, no need to lead to the same expected values of similarly-defined observables. On the other hand, we do expect, on the grounds of the zeroth law of thermodynamics, that any probe in contact with different thermal states at the same temperature reaches the same final equilibrium state—thermal equilibrium is transitive. This is exactly what happens to our accelerated extended system in the vacuum. It evolves towards the Gibbs state given by Eq. (21), which is the same equilibrium state it would have reached if it were at rest in an inertial thermal bath at temperature *T* = *T*_U_. This is all one can ask of a legitimate thermal reservoir.

It is worth stressing that the model chosen here, although simple, does constitute a genuine extended system, since it cannot be mapped onto a two free-particle system, and hence non-local correlations shall be present in the dynamics. As for the equilibrium state, even though the free-system Hamiltonian presents only two eigenvalues, the predictions extracted from it cannot emerge from a simple two-level analysis, because the corresponding eigenenergy subspaces are spanned by states with different local properties. Such a character leads the expected values of local observables, e.g., $\langle \hat s_M^j\rangle = {\mathrm{Tr}}(\hat \rho _{\mathrm{s}}\hat s_M^j)$, to be ill-determined in the two-level modeling. Our extended system captures all features used in the literature to argue against the physical reality of the Unruh temperature, with *T*_U_ = *a*/(2*π*) emerging naturally from the thermalization process observed for the spin system. Therefore, there is no reason to believe that our conclusions would not hold for more complex extended systems. In particular, a dramatic conjectured consequence is the existence of a critical acceleration above which an accelerated magnet in the vacuum would be demagnetized, something which would be hard to anticipate if it were not for the Unruh effect. This complex situation is currently under investigation.

## Methods

### Validity of the Markovian regime

**Fig. 7:**
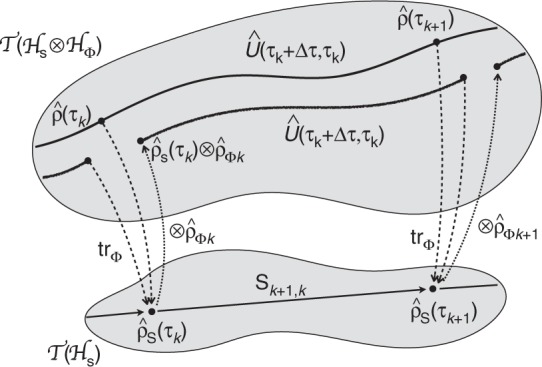
Markovian regime and the evolution of the reduced density operator. This is a schematic representation of the condition for the validity of the Markovian regime for the evolution of the open spin system. The full (unitary) evolution in the space of trace-class operators describing the universe, ${\cal{T}}$(${\cal{H}}$_s_ ⊗ ${\cal{H}}$_Φ_), must induce discrete dynamical maps S_*k*+1,*k*_ on the space of trace-class operators describing the spin system, ${\cal{T}}$(${\cal{H}}$_s_), in such a way that, for *m* ≥ *l* ≥ *k*, S_*m*,*l*_ ⋅ S_*l*,*k*_ = S_*m*,*k*_ (semigroup property)

The strategy of breaking down the long-term evolution of the (open) spin system into a sequence of *N* (≫1) time lapses Δ*τ*, such that in each time lapse, for sufficiently small coupling *q*, the spins’ evolution is well described by Eq. (10), depends on the existence of an appropriate time lapse Δ*τ* and a sequence of field states $\left\{ {\hat \rho _{{\mathrm{\Phi }}k}} \right\}_{k = 0,1, \ldots ,N - 1}$ such that

26 $${\mathrm{tr}}_{\mathrm{\Phi }}\left\{ {\hat \rho (\tau _{k + 1})} \right\} = {\mathrm{tr}}_{\mathrm{\Phi }}\left\{ {\hat U_{k + 1,k}\,{\mathrm{tr}}_{\mathrm{\Phi }}\left[ {\hat \rho (\tau _k)} \right] \otimes \hat \rho _{{\mathrm{\Phi }}k}\,\hat U_{k + 1,k}^{ - 1}} \right\},$$

where *τ*_*k*_ := *τ*_0_ + *k*Δ*τ* and $\hat U_{l,k}: = \hat U(\tau _l,\tau _k) = {\mathrm{e}}^{ - {\mathrm{i}}\hat H_0(l - k){\mathrm{\Delta }}\tau }\hat U_{\mathrm{I}}(\tau _l,\tau _k)$ (see Fig. 7). However, the condition expressed in Eq. (26) is impracticable since it assumes knowledge of the whole system evolution $\hat \rho (\tau )$. Notwithstanding, we can work with a more convenient (although stronger) condition obtained by defining the family of trace-preserving maps S_*l*,*k*_,

27 $${\mathrm{S}}_{l,k}\left( {\hat \rho _{\mathrm{s}}} \right): = {\mathrm{tr}}_{\mathrm{\Phi }}\left\{ {\hat U_{l,k}\hat \rho _{\mathrm{s}} \otimes \hat \rho _{{\mathrm{\Phi }}k}\hat U_{l,k}^{ - 1}} \right\},\quad l \ge k,$$

acting on the space of trace-class operators ${\cal{T}}\left( {{\cal{H}}_{\mathrm{s}}} \right) \ni \hat \rho _{\mathrm{s}}$ describing the spin system, and asking if there is a regime (i.e., values of Δ*τ* and $\{ \hat \rho _{{\mathrm{\Phi }}k}\} _{k = 0,1, \ldots }$) such that these maps satisfy the composition law S_*m*,*l*_ ⋅ S_*l*,*k*_ = S_*m*,*k*_, *m* ≥ *l* ≥ *k* (semigroup property). If this can be established, then Eq. (26) holds for an arbitrary simply-separable initial state $\hat \rho _0 = \hat \rho _{{\mathrm{s}}0} \otimes \hat \rho _{{\mathrm{\Phi }}0}$. We call this regime Markovian, for $\hat \rho _{\mathrm{s}}(\tau _{k + 1}) = {\mathrm{S}}_{k + 1,k}(\hat \rho _{\mathrm{s}}(\tau _k))$. Note, recalling $\hat U(\tau ,\tau _0) = {\mathrm{e}}^{ - {\mathrm{i}}\hat H_0(\tau - \tau _0)}\hat U_{\mathrm{I}}(\tau ,\tau _0)$, that ${\mathrm{S}}_{l,k}\left( {\hat \rho _{\mathrm{s}}} \right) = {\mathrm{E}}_0^{l - k}\left( {{\mathrm{S}}_{l,k}^{\mathrm{I}}\left( {\hat \rho _{\mathrm{s}}} \right)} \right)$, where ${\mathrm{E}}_0( \cdot ): = {\mathrm{e}}^{ - {\mathrm{i}}\hat H_0{\mathrm{\Delta }}\tau }( \cdot ){\mathrm{e}}^{{\mathrm{i}}\hat H_0{\mathrm{\Delta }}\tau }$ is the free evolution on ${\cal{T}}\left( {{\cal{H}}_{\mathrm{s}}} \right)$ and ${\mathrm{S}}_{l,k}^{\mathrm{I}}$ is given by Eq. (27) with $\hat U$ substituted by $\hat U_{\mathrm{I}}$.

A reasonable *ansatz* for the sequence $\left\{ {\hat \rho _{{\mathrm{\Phi }}k}} \right\}_{k = 0,1, \ldots ,N - 1}$ of field states is the one obtained by applying the analogous of Eq. (26) for obtaining the reduced density matrix describing the field state; i.e., substituting, in Eq. (26), tr_Φ_ by t*r*_s_ and $\hat \rho _{{\mathrm{\Phi }}k}$by $\hat \rho _{{\mathrm{s}}k} = \hat \rho _{\mathrm{s}}(\tau _k)$. This, together with Eq. (26), would lead to a coupled evolution of reduced density matrices $\hat \rho _{\mathrm{s}}(\tau _k)$ and $\hat \rho _{\mathrm{\Phi }}(\tau _k)$. In our case of interest, however, we expect, on physical grounds, that after some transient time—related to the time needed for the spins to exchange information via fields and the decay of the field’s correlation functions—the field state with which the spins interact continues to be well approximated by the initial stationary state, so that $\hat \rho _{{\mathrm{\Phi }}k} = \hat \rho _{{\mathrm{\Phi }}0}$ may provide a good candidate sequence. Indeed, the description put forward here is the one associated with the Markov approximation assumed in the context of open quantum systems^12,13^. There, it is well established that such an approximation furnishes a good description for the system’s reduced dynamics as long as the key elements are satisfied, namely, (i) the environment role is played by a large system (huge number of degrees of freedom) in a thermal state; (ii) the system-environment coupling can be considered weak; (iii) the environment-correlation-functions time decay must be much shorter than the system evolution time scale.

As a consequence of this approximation, S_*l*,*k*_ only depends on *l* − *k* and the composition rule then demands

28 $${\mathrm{E}}_0^{l - k} \cdot {\mathrm{S}}_{l,k}^{\mathrm{I}} = \left( {{\mathrm{E}}_0 \cdot {\mathrm{S}}_{\mathrm{I}}} \right)^{l - k},$$

where, for sufficiently small coupling *q*, ${\mathrm{S}}_{\mathrm{I}}(\hat \rho _{\mathrm{s}}): = {\mathrm{S}}_{k + 1,k}^{\mathrm{I}}(\hat \rho _{\mathrm{s}}) \approx \left( {1 - q^2{\mathrm{R}}_{{\mathrm{\Delta }}\tau }} \right)\hat \rho _{\mathrm{s}}$ can be read from the right-hand side of Eq. (10)—substituting $\hat \rho _{{\mathrm{s}}0}$ by $\hat \rho _{\mathrm{s}}$ in Eqs. (11) and (12); the linear transformation R_Δ*τ*_, acting on ${\cal{T}}\left( {{\cal{H}}_{\mathrm{s}}} \right)$, stands for the second-order term of Eq. (10) for a given time lapse Δ*τ*.

Summing up, the strategy of breaking down long-term evolution of the (open) spin system into *N* limited time steps Δ*τ*, for each of which Eq. (10) can be applied, depends on the validity of Eq. (28) for some Δ*τ*. In particular, for *n* time steps such that Eq. (10) can still be used for the time lapse *n*Δ*τ*, Eq. (28) implies

29 $${\mathrm{R}}_{n{\mathrm{\Delta }}\tau } = {\mathrm{R}}_{{\mathrm{\Delta }}\tau } + {\mathrm{E}}_0^{ - 1} \cdot {\mathrm{R}}_{(n - 1){\mathrm{\Delta }}\tau } \cdot {\mathrm{E}}_0.$$

The linear transformations E_0_ and R_Δ*τ*_ acting on the space of density matrices can be explicitly represented as 16 × 16 matrices once a basis for the spin states and an ordering of indices of $\hat \rho _{\mathrm{s}}$ are chosen. For instance, one could use the product states |±〉_A_|±〉_B_ as elements of the basis—where $\hat s_M^{\mathrm{z}}| \pm \rangle _M = \pm (1/2)| \pm \rangle _M$—, define the density-matrix elements $\rho _{\alpha \prime \beta \prime }^{\alpha \beta }: = {}_{\mathrm{B}}\langle \beta |{}_{\mathrm{A}}\langle \alpha |\hat \rho |\alpha \prime \rangle _{\mathrm{A}}|\beta \prime \rangle _{\mathrm{B}}$, and then sort these elements in a column matrix as

30 $$\hat \rho = \left( {\rho _{ + + }^{ + + }\,\,\rho _{ + - }^{ + + }\,\,\rho _{ - + }^{ + + }\,\,\rho _{ - - }^{ + + }\,\,\rho _{ + + }^{ + - }\, \ldots .\,\rho _{ - - }^{ - - }} \right)^ \top .$$

This would lead to a particular representation of E_0_ and R_Δ*τ*_. The physical conclusions are, of course, independent of the representation that is chosen.

It turns out that E_0_ and R_Δ*τ*_ assume simpler forms when density matrices are expressed in the Bell basis, formed by the elements

31 $$|{\mathrm{\Psi }}_{{\mathrm{AB}}}^{( \pm )}\rangle : = \frac{1}{{\sqrt 2 }}\left( {| + \rangle _{\mathrm{A}}| + \rangle _{\mathrm{B}} \pm | - \rangle _{\mathrm{A}}| - \rangle _{\mathrm{B}}} \right),$$

32 $$|{\mathrm{\Phi }}_{{\mathrm{AB}}}^{( \pm )}\rangle : = \frac{1}{{\sqrt 2 }}\left( {| + \rangle _{\mathrm{A}}| - \rangle _{\mathrm{B}} \pm | - \rangle _{\mathrm{A}}| + \rangle _{\mathrm{B}}} \right).$$

In this representation, E_0_ is diagonal—as in the product-state basis—and R_Δ*τ*_ is “almost diagonal”: all but 32 (out of the 240) off-diagonal terms vanish. In addition, one can explicitly check that the part of R_Δ*τ*_ associated with $\hat C_{(MN)}^{( + )}$ and $\hat D_{(MN)}^{( - )}$ commutes with the free evolution E_0_, which is not the case for the part associated with $\hat C_{(MN)}^{( - )}$ and $\hat D_{(MN)}^{( + )}$.

Considering that the Feynman correlators appearing in Eq. (10) decrease fast enough for *ξ* ≫ *d*, one can verify that for a (limited) Δ*τ* ≫ *J*^−1^, *d*, Eq. (10) assumes the asymptotic form

33 $$\begin{array}{l}{\mathrm{e}}^{{\mathrm{i}}\hat H_0{\mathrm{\Delta }}\tau }\hat \rho _{\mathrm{s}}(\tau ){\mathrm{e}}^{ - {\mathrm{i}}\hat H_0{\mathrm{\Delta }}\tau }\mathop {\sim }\limits^{{\mathrm{\Delta }}\tau \gg J^{ - 1},d} \hat \rho _{{\mathrm{s}}0} - \frac{{q^2}}{2}{\mathrm{\Delta }}\tau \mathop {\sum }\limits_{M,N \in \{ {\mathrm{A}},{\mathrm{B}}\} } \left\{ {{\mathrm{i}}\pi \tilde G_{MN}(J/2)\hat C_{(MN)}^{( + )}} \right.\\ \left. { - {\cal{P}}_{J/2}\left[ {{\mathrm{i}}\tilde G_{MN}} \right]\hat D_{(MN)}^{( - )}} \right\} + {\mathrm{H}}.{\mathrm{c}}. + {\cal{O}}(q^3),\end{array}$$

where $\tilde G_{MN}(J/2)$ and ${\cal{P}}_{J/2}[{\mathrm{i}}\tilde G_{MN}]$ are defined through Eqs. (15) and (16). Therefore, in this regime, R_Δ*τ*_ is linear in Δ*τ*, R_Δ*τ*_ ≡ Δ*τ*R_0_—see Eq. (14)—and commutes with the free evolution E_0_ (see remarks above). This is enough to guarantee that Eq. (29) is satisfied and, thus, establish that for sufficiently small *q*, the long-term evolution of the reduced spin system is given by

34 $$\hat \rho _{\mathrm{s}}(\tau _N) = {\mathrm{S}}_{N,0}\left( {\hat \rho _{{\mathrm{s}}0}} \right) = {\mathrm{e}}^{ - {\mathrm{i}}\hat H_0N{\mathrm{\Delta }}\tau }\left( {{\mathrm{e}}^{ - q^2{\mathrm{R}}_0N{\mathrm{\Delta }}\tau }\hat \rho _{{\mathrm{s}}0}} \right){\mathrm{e}}^{{\mathrm{i}}\hat H_0N{\mathrm{\Delta }}\tau }.$$

### Decaying modes and related decay rates of the spin system

Here, we present the eigenvalues *λ*_*k*_ and eigenmatrices $\hat \rho _k$ of the linear operator R_0_, appearing in the long-term evolution above, in terms of ${\mathrm{i}}\tilde G_{MN}(J/2)$, $P_{J/2}[{\mathrm{i}}\tilde G_{MN}]$, and the Bell states defined in Eqs. (31) and (32). First, we define the following real quantities:

35 $$\begin{array}{*{20}{l}} {\alpha _{\mathrm{s}}^ \pm } \hfill & {: = } \hfill & {\frac{\pi }{2}\left[ {{\mathrm{Re}}\left\{ {{\mathrm{i}}\tilde G_{{\mathrm{AA}}}(J/2)} \right\} + {\mathrm{Re}}\left\{ {{\mathrm{i}}\tilde G_{{\mathrm{BB}}}(J/2)} \right\}} \right]} \hfill \\ {} \hfill & {} \hfill & { \pm \,\frac{1}{2}\left[ {{\mathrm{Im}}\left\{ {{\cal{P}}_{J/2}\left[ {{\mathrm{i}}\tilde G_{{\mathrm{AA}}}} \right]} \right\} + {\mathrm{Im}}\left\{ {{\cal{P}}_{J/2}\left[ {{\mathrm{i}}\tilde G_{{\mathrm{BB}}}} \right]} \right\}} \right],} \hfill \end{array}$$

36 $$\begin{array}{*{20}{l}} {{\mathrm{\Delta }}\alpha _{\mathrm{s}}^ \pm } \hfill & {: = } \hfill & {\frac{\pi }{2}\left[ {{\mathrm{Re}}\left\{ {{\mathrm{i}}\tilde G_{{\mathrm{A}}A}(J/2)} \right\} - {\mathrm{Re}}\left\{ {{\mathrm{i}}\tilde G_{{\mathrm{BB}}}(J/2)} \right\}} \right]} \hfill \\ {} \hfill & {} \hfill & { \pm \frac{1}{2}\left[ {{\mathrm{Im}}\left\{ {{\cal{P}}_{J/2}\left[ {{\mathrm{i}}\tilde G_{{\mathrm{AA}}}} \right]} \right\} - {\mathrm{Im}}\left\{ {{\cal{P}}_{J/2}\left[ {{\mathrm{i}}\tilde G_{{\mathrm{BB}}}} \right]} \right\}} \right],} \hfill \end{array}$$

37 $$\begin{array}{*{20}{l}} {\alpha _{\mathrm{i}}^\pm } \hfill & {: = } \hfill & {\pi {\mathrm{Re}}\left\{ {{\mathrm{i}}\tilde G_{{\mathrm{AB}}}(J/2)} \right\} \pm {\mathrm{Im}}\left\{ {{\cal{P}}_{J/2}\left[ {{\mathrm{i}}\tilde G_{{\mathrm{AB}}}} \right]} \right\},} \hfill \end{array}$$

38 $$\begin{array}{*{20}{l}} {\beta _{\mathrm{s}}^ \pm } \hfill & {: = } \hfill & {\frac{\pi }{2}\left[ {{\mathrm{Im}}\left\{ {{\mathrm{i}}\tilde G_{{\mathrm{AA}}}(J/2)} \right\} + {\mathrm{Im}}\left\{ {{\mathrm{i}}\tilde G_{{\mathrm{BB}}}(J/2)} \right\}} \right]} \hfill\\ & &\pm \frac{1}{2}\left[ {{\mathrm{Re}}\left\{ {{\cal{P}}_{J/2}\left[ {{\mathrm{i}}\tilde G_{{\mathrm{AA}}}} \right]} \right\} + {\mathrm{Re}}\left\{ {{\cal{P}}_{J/2}\left[ {{\mathrm{i}}\tilde G_{{\mathrm{BB}}}} \right]} \right\}} \right],\end{array}$$

39 $$\begin{array}{ccccc}\\ {\mathrm{\Delta }}\beta _{\mathrm{s}}^ \pm : = \frac{\pi }{2}\left[ {{\mathrm{Im}}\left\{ {{\mathrm{i}}\tilde G_{{\mathrm{AA}}}(J/2)} \right\} - {\mathrm{Im}}\left\{ {{\mathrm{i}}\tilde G_{{\mathrm{BB}}}(J/2)} \right\}} \right]\\ \\ {\hskip 50pt}\pm \frac{1}{2}\left[ {{\mathrm{Re}}\left\{ {{\cal{P}}_{J/2}\left[ {{\mathrm{i}}\tilde G_{{\mathrm{AA}}}} \right]} \right\} - {\mathrm{Re}}\left\{ {{\cal{P}}_{J/2}\left[ {{\mathrm{i}}\tilde G_{{\mathrm{BB}}}} \right]} \right\}} \right],\\ \end{array}$$

40 $$\beta _{\mathrm{i}}^ \pm : = \pi {\mathrm{Im}}\left\{ {{\mathrm{i}}\tilde G_{{\mathrm{AB}}}(J/2)} \right\} \pm {\mathrm{Re}}\left\{ {{\cal{P}}_{J/2}\left[ {{\mathrm{i}}\tilde G_{{\mathrm{AB}}}} \right]} \right\},$$

from which all ${\mathrm{i}}\tilde G_{MN}(J/2)$ and ${\cal{P}}_{J/2}\left[ {{\mathrm{i}}\tilde G_{MN}} \right]$ can be reconstructed. Also, let *r*_0_, *r*_±_ be the roots of the polynomial

41 $$\mathrm{P}(r) :=	 \ r^3 - 2(\alpha _{\mathrm{s}}^ - + 2\alpha _{\mathrm{s}}^ + )r^2 + 4\left[ {(\alpha _{\mathrm{s}}^ + )^2 + 2\alpha _{\mathrm{s}}^ - \alpha _{\mathrm{s}}^ + + (\beta _{\mathrm{i}}^ - )^2 - {\mathrm{\Delta }}\alpha _{\mathrm{s}}^ - {\mathrm{\Delta }}\alpha _{\mathrm{s}}^ + } \right]r \\ 	- 8\alpha _{\mathrm{s}}^ - \left[ {(\alpha _{\mathrm{s}}^ + )^2 + (\beta _{\mathrm{i}}^ - )^2} \right] + 8\alpha _{\mathrm{s}}^ + {\mathrm{\Delta }}\alpha _{\mathrm{s}}^ - {\mathrm{\Delta }}\alpha _{\mathrm{s}}^ + ,$$

ordered, by convention, in such a way that they are continuous functions of ${\mathrm{\Delta }}\alpha _{\mathrm{s}}^ + {\mathrm{\Delta }}\alpha _{\mathrm{s}}^ -$ and, for small enough ${\mathrm{\Delta }}\alpha _{\mathrm{s}}^ + {\mathrm{\Delta }}\alpha _{\mathrm{s}}^ -$, $r_0 \approx 2\alpha _{\mathrm{s}}^ - + O({\mathrm{\Delta }}\alpha _{\mathrm{s}}^ + {\mathrm{\Delta }}\alpha _{\mathrm{s}}^ - )$, $r_ \pm \approx 2(\alpha _{\mathrm{s}}^ + \pm i\beta _{\mathrm{i}}^ - ) + O({\mathrm{\Delta }}\alpha _{\mathrm{s}}^ + {\mathrm{\Delta }}\alpha _{\mathrm{s}}^ - )$. Then, the eigenvalues and eigenmatrices are:

42 $$\lambda _1 = 0,$$

43 $$\begin{array}{ccccc} \hat \rho _1 = \frac{1}{{2\left[ {(\alpha _{\mathrm{s}}^ + )^2 - (\alpha _{\mathrm{i}}^ + )^2 + \alpha _{\mathrm{s}}^ + \alpha _{\mathrm{s}}^ - - \alpha _{\mathrm{i}}^ + \alpha _{\mathrm{i}}^ - } \right]}}\left\{ {\left[ {(\alpha _{\mathrm{s}}^ + )^2 - (\alpha _{\mathrm{i}}^ + )^2} \right]\left( {|{\mathrm{\Psi }}_{{\mathrm{AB}}}^{( + )}\rangle \langle {\mathrm{\Psi }}_{{\mathrm{AB}}}^{( + )}| + |{\mathrm{\Psi }}_{{\mathrm{AB}}}^{( - )}\rangle \langle {\mathrm{\Psi }}_{{\mathrm{AB}}}^{( - )}|} \right)} \right. \\ \ \ \ \ \left. { + (\alpha _{\mathrm{s}}^ + + \alpha _{\mathrm{i}}^ + )(\alpha _{\mathrm{s}}^ - - \alpha _{\mathrm{i}}^ - )|{\mathrm{\Phi }}_{{\mathrm{AB}}}^{( - )}\rangle \langle {\mathrm{\Phi }}_{{\mathrm{AB}}}^{( - )}| + (\alpha _{\mathrm{s}}^ + - \alpha _{\mathrm{i}}^ + )(\alpha _{\mathrm{s}}^ - + \alpha _{\mathrm{i}}^ - )|{\mathrm{\Phi }}_{{\mathrm{AB}}}^{( + )}\rangle \langle {\mathrm{\Phi }}_{{\mathrm{AB}}}^{( + )}|} \right\};\\ \end{array}$$

44 $$\lambda _2 = 2\alpha _{\mathrm{s}}^ + + \alpha _{\mathrm{s}}^ - + \sqrt {(\alpha _{\mathrm{s}}^ - )^2 + 4\alpha _{\mathrm{i}}^ + (\alpha _{\mathrm{i}}^ + + \alpha _{\mathrm{i}}^ - )} ,$$

45 $$\begin{array}{ccccc} \hat \rho _2 = |{\mathrm{\Psi }}_{{\mathrm{AB}}}^{( + )}\rangle \langle {\mathrm{\Psi }}_{{\mathrm{AB}}}^{( + )}| + |{\mathrm{\Psi }}_{{\mathrm{AB}}}^{( - )}\rangle \langle {\mathrm{\Psi }}_{{\mathrm{AB}}}^{( - )}| - \left[ {1 + \frac{{\sqrt {(\alpha _{\mathrm{s}}^ - )^2 + 4\alpha _{\mathrm{i}}^ + (\alpha _{\mathrm{i}}^ + + \alpha _{\mathrm{i}}^ - )} - \alpha _{\mathrm{s}}^ - }}{{2\alpha _{\mathrm{i}}^ + }}} \right]|{\mathrm{\Phi }}_{{\mathrm{AB}}}^{( + )}\rangle \langle {\mathrm{\Phi }}_{{\mathrm{AB}}}^{( + )}|\\ \\ - \left[ {1 - \frac{{\sqrt {(\alpha _{\mathrm{s}}^ - )^2 + 4\alpha _{\mathrm{i}}^ + (\alpha _{\mathrm{i}}^ + + \alpha _{\mathrm{i}}^ - )} - \alpha _{\mathrm{s}}^ - }}{{2\alpha _{\mathrm{i}}^ + }}} \right]|{\mathrm{\Phi }}_{{\mathrm{AB}}}^{( - )}\rangle \langle {\mathrm{\Phi }}_{{\mathrm{AB}}}^{( - )}|; \end{array}$$

46 $$\lambda _3 = 2\alpha _{\mathrm{s}}^ + + \alpha _{\mathrm{s}}^ - - \sqrt {(\alpha _{\mathrm{s}}^ - )^2 + 4\alpha _{\mathrm{i}}^ + (\alpha _{\mathrm{i}}^ + + \alpha _{\mathrm{i}}^ - )} ,$$

47 $$\begin{array}{ccccc} \hat \rho _3 = |{\mathrm{\Psi }}_{{\mathrm{AB}}}^{( + )}\rangle \langle {\mathrm{\Psi }}_{{\mathrm{AB}}}^{( + )}| + |{\mathrm{\Psi }}_{{\mathrm{AB}}}^{( - )}\rangle \langle {\mathrm{\Psi }}_{{\mathrm{AB}}}^{( - )}| - \left[ {1 - \frac{{\sqrt {(\alpha _{\mathrm{s}}^ - )^2 + 4\alpha _{\mathrm{i}}^ + (\alpha _{\mathrm{i}}^ + + \alpha _{\mathrm{i}}^ - )} + \alpha _{\mathrm{s}}^ - }}{{2\alpha _{\mathrm{i}}^ + }}} \right]|{\mathrm{\Phi }}_{{\mathrm{AB}}}^{( + )}\rangle \langle {\mathrm{\Phi }}_{{\mathrm{AB}}}^{( + )}|\\ \\ - \left[ {1 + \frac{{\sqrt {(\alpha _{\mathrm{s}}^ - )^2 + 4\alpha _{\mathrm{i}}^ + (\alpha _{\mathrm{i}}^ + + \alpha _{\mathrm{i}}^ - )} + \alpha _{\mathrm{s}}^ - }}{{2\alpha _{\mathrm{i}}^ + }}} \right]|{\mathrm{\Phi }}_{{\mathrm{AB}}}^{( - )}\rangle \langle {\mathrm{\Phi }}_{{\mathrm{AB}}}^{( - )}|;\\ \end{array}$$

48 $$\lambda _4 = 2\alpha _{\mathrm{s}}^ - = \lambda _5,$$

49 $$\hat \rho _4 = |{\mathrm{\Psi }}_{{\mathrm{AB}}}^{( + )}\rangle \langle {\mathrm{\Psi }}_{{\mathrm{AB}}}^{( + )}| - |{\mathrm{\Psi }}_{{\mathrm{AB}}}^{( - )}\rangle \langle {\mathrm{\Psi }}_{{\mathrm{AB}}}^{( - )}|,$$

50 $$\hat \rho _5 = {\mathrm{i}}|{\mathrm{\Psi }}_{{\mathrm{AB}}}^{( + )}\rangle \langle {\mathrm{\Psi }}_{{\mathrm{AB}}}^{( - )}| - {\mathrm{i}}|{\mathrm{\Psi }}_{{\mathrm{AB}}}^{( - )}\rangle \langle {\mathrm{\Psi }}_{{\mathrm{AB}}}^{( + )}|;$$

51 $$\lambda _6 = r_0,$$

52 $${\hat \rho _6 = |{\mathrm{\Psi }}_{{\mathrm{AB}}}^{( + )}\rangle \langle {\mathrm{\Psi }}_{{\mathrm{AB}}}^{( - )}| + |{\mathrm{\Psi }}_{{\mathrm{AB}}}^{( - )}\rangle \langle {\mathrm{\Psi }}_{{\mathrm{AB}}}^{( + )}| + 2{\mathrm{\Delta }}\alpha _{\mathrm{s}}^ - \left[ {\frac{{|{\mathrm{\Phi }}_{{\mathrm{AB}}}^{( + )}\rangle \langle {\mathrm{\Phi }}_{{\mathrm{AB}}}^{( - )}|}}{{r_0 - 2\left( {\alpha _{\mathrm{s}}^ + + {\mathrm{i}}\beta _{\mathrm{i}}^ - } \right)}} + \frac{{|{\mathrm{\Phi }}_{{\mathrm{AB}}}^{( - )}\rangle \langle {\mathrm{\Phi }}_{{\mathrm{AB}}}^{( + )}|}}{{r_0 - 2\left( {\alpha _{\mathrm{s}}^ + - {\mathrm{i}}\beta _{\mathrm{i}}^ - } \right)}}} \right];}$$

53 $$\lambda _7 = \alpha _{\mathrm{s}}^ + + \alpha _{\mathrm{s}}^ - - \alpha _{\mathrm{i}}^ + + {\mathrm{i}}(\beta _{\mathrm{s}}^ + - \beta _{\mathrm{s}}^ - + \beta _{\mathrm{i}}^ - ) = \lambda _8^ \ast = \lambda _9 = \lambda _{10}^ \ast ,$$

54 $$\hat \rho _7 = |{\mathrm{\Psi }}_{{\mathrm{AB}}}^{( - )}\rangle \langle {\mathrm{\Phi }}_{{\mathrm{AB}}}^{( - )}| = \hat \rho _8^\dagger ,$$

55 $$\hat \rho _9 = |{\mathrm{\Psi }}_{{\mathrm{AB}}}^{( + )}\rangle \langle {\mathrm{\Phi }}_{{\mathrm{AB}}}^{( - )}| = \hat \rho _{10}^\dagger ;$$

56 $$\lambda _{11} = \alpha _{\mathrm{s}}^ + + \alpha _{\mathrm{s}}^ - + \alpha _{\mathrm{i}}^ + + {\mathrm{i}}\left( {\beta _{\mathrm{s}}^ + - \beta _{\mathrm{s}}^ - - \beta _{\mathrm{i}}^ - } \right) = \lambda _{12}^ \ast = \lambda _{13} = \lambda _{14}^ \ast ,$$

57 $$\hat \rho _{11} = |{\mathrm{\Psi }}_{{\mathrm{AB}}}^{( - )}\rangle \langle {\mathrm{\Phi }}_{{\mathrm{AB}}}^{( + )}| = \hat \rho _{12}^\dagger ,$$

58 $$\hat \rho _{13} = |{\mathrm{\Psi }}_{{\mathrm{AB}}}^{( + )}\rangle \langle {\mathrm{\Phi }}_{{\mathrm{AB}}}^{( + )}| = \hat \rho _{14}^\dagger ;$$

59 $$\lambda _{15} = r_ - ,$$

60 $${\hat \rho _{15} = {\mathrm{\Delta }}\alpha _{\mathrm{s}}^ + \left[ {|{\mathrm{\Psi }}_{{\mathrm{AB}}}^{( + )}\rangle \langle {\mathrm{\Psi }}_{{\mathrm{AB}}}^{( - )}| + |{\mathrm{\Psi }}_{{\mathrm{AB}}}^{( - )}\rangle \langle {\mathrm{\Psi }}_{{\mathrm{AB}}}^{( + )}|} \right] + 2{\mathrm{\Delta }}\alpha _{\mathrm{s}}^ + {\mathrm{\Delta }}\alpha _{\mathrm{s}}^ - \left[ {\frac{{|{\mathrm{\Phi }}_{{\mathrm{AB}}}^{( + )}\rangle \langle {\mathrm{\Phi }}_{{\mathrm{AB}}}^{( - )}|}}{{r_ - - 2(\alpha _{\mathrm{s}}^ + + {\mathrm{i}}\beta _{\mathrm{i}}^ - )}} + \frac{{|{\mathrm{\Phi }}_{{\mathrm{AB}}}^{( - )}\rangle \langle {\mathrm{\Phi }}_{{\mathrm{AB}}}^{( + )}|}}{{r_ - - 2(\alpha _{\mathrm{s}}^ + - {\mathrm{i}}\beta _{\mathrm{i}}^ - )}}} \right];}$$

61 $$\lambda _{16} = r_ + ,$$

62 $${\hat \rho _{16} = {\mathrm{\Delta }}\alpha _{\mathrm{s}}^ + \left[ {|{\mathrm{\Psi }}_{{\mathrm{AB}}}^{( + )}\rangle \langle {\mathrm{\Psi }}_{{\mathrm{AB}}}^{( - )}| + |{\mathrm{\Psi }}_{{\mathrm{AB}}}^{( - )}\rangle \langle {\mathrm{\Psi }}_{{\mathrm{AB}}}^{( + )}|} \right] + 2{\mathrm{\Delta }}\alpha _{\mathrm{s}}^ + {\mathrm{\Delta }}\alpha _{\mathrm{s}}^ - \left[ {\frac{{|{\mathrm{\Phi }}_{{\mathrm{AB}}}^{( + )}\rangle \langle {\mathrm{\Phi }}_{{\mathrm{AB}}}^{( - )}|}}{{r_ + - 2(\alpha _{\mathrm{s}}^ + + {\mathrm{i}}\beta _{\mathrm{i}}^ - )}} + \frac{{|{\mathrm{\Phi }}_{{\mathrm{AB}}}^{( - )}\rangle \langle {\mathrm{\Phi }}_{{\mathrm{AB}}}^{( + )}|}}{{r_ + - 2\left( {\alpha _{\mathrm{s}}^ + - {\mathrm{i}}\beta _{\mathrm{i}}^ - } \right)}}} \right].}$$

We note that in case ${\mathrm{\Delta }}\alpha _{\mathrm{s}}^ + {\mathrm{\Delta }}\alpha _{\mathrm{s}}^ - = 0$—which encompasses both scenarios analyzed in this work (equal and different spins’ proper accelerations)—Eqs. (51), (52), (59)–(62) can be conveniently reduced to $\lambda _6 = 2\alpha _{\mathrm{s}}^ -$, $\hat \rho _6 = |{\mathrm{\Psi }}_{{\mathrm{AB}}}^{( + )}\rangle \langle {\mathrm{\Psi }}_{{\mathrm{AB}}}^{( - )}| + |{\mathrm{\Psi }}_{{\mathrm{AB}}}^{( - )}\rangle \langle {\mathrm{\Psi }}_{{\mathrm{AB}}}^{( + )}|$, $\lambda _{15} = 2(\alpha _{\mathrm{s}}^ + - {\mathrm{i}}\beta _{\mathrm{i}}^ - ) = \lambda _{16}^ \ast$, $\hat \rho _{15} = |{\mathrm{\Phi }}_{{\mathrm{AB}}}^{( - )}\rangle \langle {\mathrm{\Phi }}_{{\mathrm{AB}}}^{( + )}| = \hat \rho _{{\mathrm{16}}}^\dagger$.

Note that the “mode” associated with the null eigenvalue, $\hat \rho _1$—which gives the final equilibrium state in the long-term evolution of the spin system; see Eq. (17)—is diagonal in the Bell basis. Therefore, regardless the form of the Feynman correlator *G*_*MN*_—provided Re(*λ*_*k*≠1_) > 0—the spin system evolves to a statistical mixture of Bell states, with populations which depend on the specific form of *G*_*MN*_. Notice from Eq. (43), however, that $|{\mathrm{\Psi }}_{{\mathrm{AB}}}^{( \pm )}\rangle$ are equally populated regardless the form of *G*_*MN*_, which means that the equilibrium state is also a statistical mixture of the separable states |+〉_A_|+〉_B_ and |−〉_A_|−〉_B_. The same is not true for $|{\mathrm{\Phi }}_{{\mathrm{AB}}}^{( \pm )}\rangle$: depending on *G*_*MN*_, the final equilibrium state may preserve correlations between |+〉_A_|−〉_B_ and |−〉_A_|+〉_B_. These results can be summarized as follows: in general, the spin system will loose coherence in any basis which diagonalizes, simultaneously, the free Hamiltonian $\hat H_0$ and the total spin ${\hat{\mathrm{S}}}^2: = \mathop {\sum }\limits_{j \in \{ {\mathrm{x}},{\mathrm{y}},{\mathrm{z}}\} } \left( {\hat s_{\mathrm{A}}^j + \hat s_{\mathrm{B}}^j} \right)^2$.

### Transformed Feynman correlators and their principal values

Here, we calculate the quantities ${\mathrm{i}}\tilde G_{MN}$ and $P_a[{\mathrm{i}}\tilde G_{MN}]$ which completely determine the long-term evolution of the spin system through Eqs. (17) and (35)–(62). Treating first the case of spins with the equal proper accelerations, Eq. (19) leads to

63 $$\sigma (x_{\mathrm{A}},x_{\mathrm{A}}^\prime ) = \sigma (x_{\mathrm{B}},x_{\mathrm{B}}^\prime ) = - \frac{4}{{a^2}}\left[ {{\mathrm{sinh}}\left( {\frac{{a\xi }}{2}} \right)} \right]^2,$$

64 $$\sigma \left( {x_{\mathrm{A}},x_{\mathrm{B}}^\prime } \right) = \sigma \left( {x_{\mathrm{B}},x_{\mathrm{A}}^\prime } \right) = - \frac{4}{{a^2}}\left[ {{\mathrm{sinh}}\left( {\frac{{a\xi }}{2}} \right)} \right]^2 + d^2,$$

where *ξ* = *τ* − *τ*′. Substituting these expressions into Eq. (18) and using that i*G*_*MN*_(*ξ*) = *θ*(*ξ*)*W*(*x*_*M*_, *x*′_*N*_) + *θ*(−*ξ*)*W*(*x*′_*N*_, *x*_*M*_)—where *θ*(*ξ*) is the Heaviside step function—we obtain, using Eqs. (15) and (16), the quantities

65 $${\mathrm{i}}\tilde G_{{\mathrm{AA}}}(\omega ) = {\mathrm{i}}\tilde G_{{\mathrm{BB}}}(\omega ) = \frac{1}{{8\pi ^2}}\left[ {\omega {\mathrm{coth}}\left( {\frac{{\pi \omega }}{a}} \right) - {\mathrm{i}}\begin{array}{*{20}{c}} {{\mathrm{lim}}} \\ {\varepsilon \to 0 + } \end{array}\frac{1}{\varepsilon }} \right],$$

66 $${\mathrm{i}}\tilde G_{{\mathrm{AB}}}(\omega ) = \frac{{{\mathrm{sin}}\left( {\frac{{2\omega }}{a}{\mathrm{sinh}}^{ - 1}\left( {\frac{{ad}}{2}} \right)} \right)}}{{4\pi ^2d\sqrt {4 + a^2d^2} }}\left[ {{\mathrm{coth}}\left( {\frac{{\pi \omega }}{a}} \right) - {\mathrm{icot}}\left( {\frac{{2\omega }}{a}{\mathrm{sinh}}^{ - 1}\left( {\frac{{ad}}{2}} \right)} \right)} \right].$$

The complex infinite in Eq. (65) is a consequence of the “too-singular” (ultraviolet) behavior of *G*_*MM*_(*ξ*) at the vertex of the light cone. One could “smooth” this singularity by smearing out the position of the spins. However, this is not necessary for our purposes since it does not affect any physical result [notice, from Eqs. (35)–(40) that this divergence only contributes—equally—to $\beta _{\mathrm{s}}^ \pm$, which, by their turn, only appear in Eqs. (53) and (56), in such a way that the divergences cancel out]. Then, with the help of Eq. (4.115.8) of ref. ^14^, we can calculate $P_{J/2}\left[ {{\mathrm{i}}\tilde G_{{\mathrm{AA}}}} \right] = P_{J/2}\left[ {{\mathrm{i}}\tilde G_{{\mathrm{BB}}}} \right]$ and $P_{J/2}\left[ {{\mathrm{i}}\tilde G_{{\mathrm{AB}}}} \right]$—the former being obtained from the limit *d* → 0_+_ of the latter:

67 $$P_{J/2}\left[ {{\mathrm{i}}\tilde G_{{\mathrm{AA}}}} \right] = P_{J/2}\left[ {{\mathrm{i}}\tilde G_{{\mathrm{BB}}}} \right] = \frac{J}{{8\pi ^2}}\left( - \lim_{\varepsilon \to 0_+}{\mathrm{ln}}\varepsilon + {\mathrm{i}}\frac{\pi }{2} \right),$$

68 $$P_{J/2}\left[ {{\mathrm{i}}\tilde G_{{\mathrm{AB}}}} \right] = \frac{1}{{4\pi ^2d\sqrt {4 + a^2d^2} }}\left[ {F\left( {\frac{J}{{2a}},{\mathrm{sinh}}^{ - 1}\left( {\frac{{ad}}{2}} \right)} \right) + {\mathrm{i}}\pi {\mathrm{sin}}\left( {\frac{J}{a}{\mathrm{sinh}}^{ - 1}\left( {\frac{{ad}}{2}} \right)} \right)} \right],$$

where

69 $$\begin{array}{*{20}{l}} {F(x,y)} 	:= {\frac{1}{x} + \pi {\mathrm{cos}}\left( {2xy} \right){\mathrm{coth}}\left( {\pi x} \right) - 2x\mathop {\sum }\limits_{n = 1}^\infty \frac{{{\mathrm{e}}^{ - 2ny}}}{{n^2 + x^2}}} \hfill \\ 	 = \hfill { - \frac{1}{x} + \pi {\mathrm{cos}}\left( {2xy} \right){\mathrm{coth}}\left( {\pi x} \right) - {\mathrm{Re}}\left\{ {{\mathrm{i}}\,{\mathrm{e}}^{2{\mathrm{i}}xy}B_{{\mathrm{e}}^{ - 2y}}\left( {1 + {\mathrm{i}}x,0} \right)} \right\}} \hfill \end{array}$$

and $B_z(x,y): = {\int}_0^z {dt} \,t^{x - 1}(1 - t)^{y - 1}$ is the incomplete Euler *β* function. Again, a divergence related to the singular behavior of *G*_*MM*_(*ξ*) at *ξ* = 0 appears in Eq. (67). This time, however, it is not obvious that this divergence will bear no consequence on physical results. And in fact, although the main features of the spins’ evolution (the final equilibrium state and relaxation/decoherence time scales) are completely oblivious to such a divergence, some transient observables (e.g., the frequency of oscillation of some decaying modes) do depend on the real part of $P_{J/2}\left[ {{\mathrm{i}}\tilde G_{MM}} \right]$ [see, again, Eqs. (38), (53), and (56)]. The equations above completely determine *λ*_*k*_ and $\hat \rho _k$ appearing in the long-term evolution of the spin system in the case of equal proper accelerations—see Eqs. (17) and (35)–(62).

In the case of spins with different proper accelerations, substituting the spins’ worldlines into Eq. (19), Eqs. (63) and (64) get replaced by:

70 $$\sigma \left( {x_{\mathrm{A}},x_{\mathrm{A}}^\prime } \right) = \frac{{\sigma \left( {x_{\mathrm{B}},x_{\mathrm{B}}^\prime } \right)}}{{(1 + ad)^2}} = - \frac{4}{{a^2}}\left[ {{\mathrm{sinh}}\left( {\frac{{a\xi }}{2}} \right)} \right]^2,$$

71 $$\sigma \left( {x_{\mathrm{A}},x_{\mathrm{B}}^\prime } \right) = \sigma \left( {x_{\mathrm{B}},x_{\mathrm{A}}^\prime } \right) = - \frac{{4(1 + ad)}}{{a^2}}\left[ {{\mathrm{sinh}}\left( {\frac{{a\xi }}{2}} \right)} \right]^2 + d^2.$$

Applying the same procedure above to these results (recalling that $u_{\mathrm{A}}^0 = 1$ and $u_{\mathrm{B}}^0 = 1/(1 + ad)$), we obtain

72 $${\mathrm{i}}\tilde G_{{\mathrm{AA}}}(\omega ) = {\mathrm{i}}\tilde G_{{\mathrm{BB}}}(\omega ) = \frac{1}{{8\pi ^2}}\left[ {\omega {\mathrm{coth}}\left( {\frac{{\pi \omega }}{a}} \right) - {\mathrm{i}}\lim_{\varepsilon \to 0 + }\frac{1}{\varepsilon }} \right],$$

73 $${{\mathrm{i}}\tilde G_{{\mathrm{AB}}}(\omega ) = \frac{{(1 + ad){\mathrm{sin}}\left( {\frac{{2\omega }}{a}{\mathrm{sinh}}^{ - 1}\left( {\frac{{ad}}{{2\sqrt {1 + ad} }}} \right)} \right)}}{{4\pi ^2d(2 + ad)}}\left[ {{\mathrm{coth}}\left( {\frac{{\pi \omega }}{a}} \right) - {\mathrm{icot}}\left( {\frac{{2\omega }}{a}{\mathrm{sinh}}^{ - 1}\left( {\frac{{ad}}{{2\sqrt {1 + ad} }}} \right)} \right)} \right],}$$

74 $$P_{J/2}\left[ {{\mathrm{i}}\tilde G_{{\mathrm{AA}}}} \right] = P_{J/2}\left[ {{\mathrm{i}}\tilde G_{{\mathrm{BB}}}} \right] = \frac{J}{{8\pi ^2}}\left( { - \lim_{\varepsilon \to 0_ + }{\mathrm{ln}}\varepsilon + {\mathrm{i}}\frac{\pi }{2}} \right),$$

75 $${P_{J/2}\left[ {{\mathrm{i}}\tilde G_{{\mathrm{AB}}}} \right] = \frac{{(1 + ad)}}{{4\pi ^2d(2 + ad)}} \times \left[ {F\left( {\frac{J}{{2a}},{\mathrm{sinh}}^{ - 1}\left( {\frac{{ad}}{{2\sqrt {1 + ad} }}} \right)} \right) + {\mathrm{i}}\pi {\mathrm{sin}}\left( {\frac{J}{a}{\mathrm{sinh}}^{ - 1}\left( {\frac{{ad}}{{2\sqrt {1 + ad} }}} \right)} \right)} \right],}$$

where *F* is still given by Eq. (69). Again, these quantities determine all the eigenvalues *λ*_*k*_ and eigenmatrices $\hat \rho _k$ which govern the long-term evolution of the spin system, now in the case of different proper accelerations.

## Data Availability

The data that support the findings of this study are available from the corresponding author upon reasonable request.

## Code availability

Numerical codes used to generate figures can be made available from the corresponding author upon reasonable request.
